# Supplementary figures and images for: Life history optimisation drives latitudinal gradients and responses to global change in marine fishes
Source: PLoS Biol. 2023 May 25;21(5):e3002114. doi: 10.1371/journal.pbio.3002114 (PMC10212075; doi:10.1371/journal.pbio.3002114)

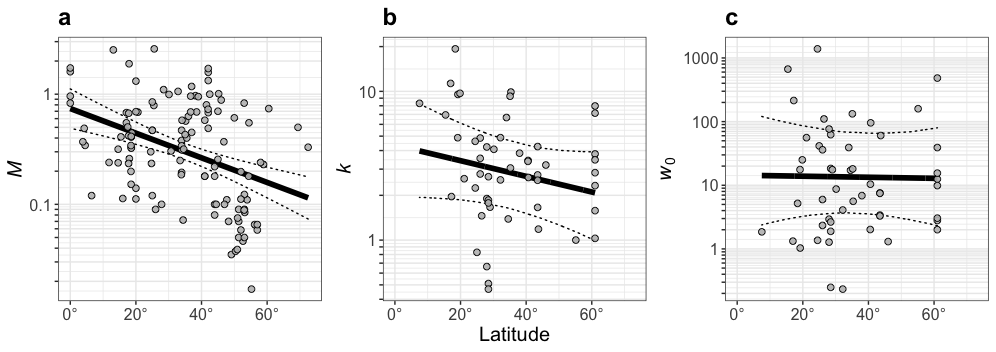

Supplement: S1 Fig — The grey points show data points. The solid lines show models’ predictions, and the dashed lines show the 95% credible intervals. (a) M as a function of latitude (Ln(M) = a + b * latitude (absolute value), where a = −0.31 (95% CI: −0.82 to 0.20) and b = −0.02 (95% CI: −0.04 to −0.01); S1 Table). The underlying data for this panel can be found in S3 Data. (b) k as a function of latitude (Ln(k) = a + b * latitude (absolute value), where a = 1.47 (95% CI: 0.66 to 2.30) and b = −0.01 (95% CI: −0.03 to 0.01); S1 Table). (c) w0 as a function of latitude (Ln(w0) = a + b * latitude (absolute value), where a = 2.67 (95% CI: 0.63 to 5.05) and b = 0.00 (95% CI: −0.05 to 0.04); S1 Table). The underlying data for panels (b) and (c) can be found in S4 Data. (TIFF) [file pbio.3002114.s001.tiff]

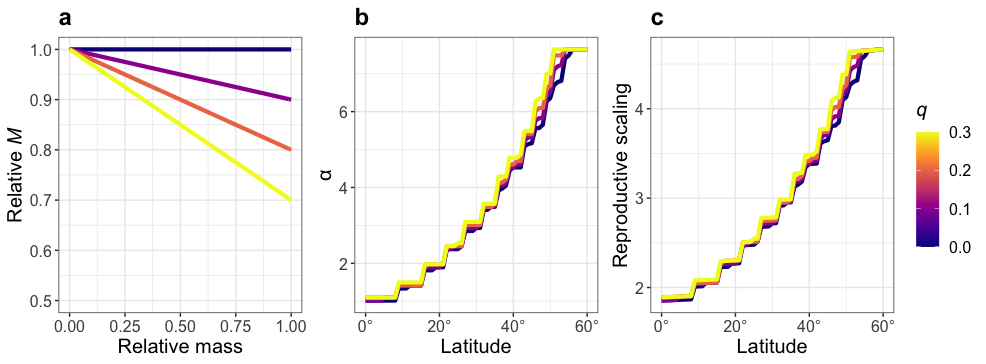

Supplement: S2 Fig — (a) Relative mortality rate M as a function of relative mass. (b) Optimal age at maturity (α) across latitudes. (c) Reproductive scaling across latitudes. The colours show the level of size dependency in mortality (q), going from size independent (purple) to strongly size dependent (yellow). Predictions underlying this figure assume changes in fecundity (F) across latitudes following the Equation in Fig 2 and S1 Table. (TIFF) [file pbio.3002114.s002.tiff]

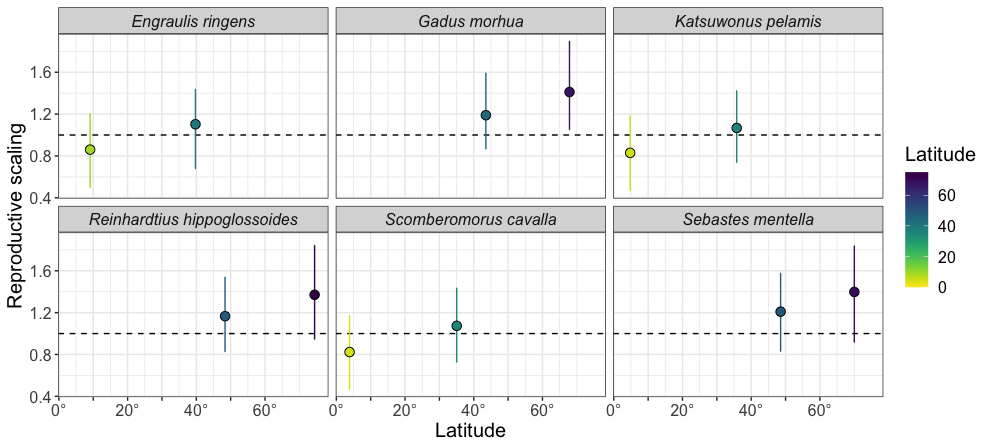

Supplement: S3 Fig — Reproductive scaling is shown for the highest and lowest latitude populations for species with reproductive data spanning >20° latitude. The error bars show the 95% credible intervals, and the colours indicate absolute latitude. The dashed black line shows reproductive isometry. The reproductive scaling for Engraulis ringens is 0.86 (95% CI: 0.50 to 1.20) and 1.10 (95% CI: 0.68 to 1.44) at absolute latitudes of 9° and 40°, respectively. The reproductive scaling for Gadus morhua is 1.19 (95% CI: 0.87 to 1.59) and 1.41 (95% CI: 1.05 to 1.90) at absolute latitudes of 43.5° and 68°, respectively. The reproductive scaling for Katsuwonus pelamis is 0.83 (95% CI: 0.47 to 0.18) and 1.07 (95% CI: 0.74 to 1.42) at absolute latitudes of 5° and 36°, respectively. The reproductive scaling for Reinhardtius hippoglossoides is 1.17 (95% CI: 0.83 to 1.54) and 1.37 (95% CI: 0.95 to 1.84) at absolute latitudes of 48° and 74.5°, respectively. The reproductive scaling for Scomberomorus cavalla is 0.82 (95% CI: 0.46 to 1.17) and 1.07 (95% CI: 0.73 to 1.44) at absolute latitudes of 4° and 35°, respectively. The reproductive scaling for Sebastes mentella is 1.21 (95% CI: 0.83 to 1.58) and 1.40 (95% CI: 0.92 to 1.84) at absolute latitudes of 48.5° and 70°, respectively. (TIFF) [file pbio.3002114.s003.tiff]

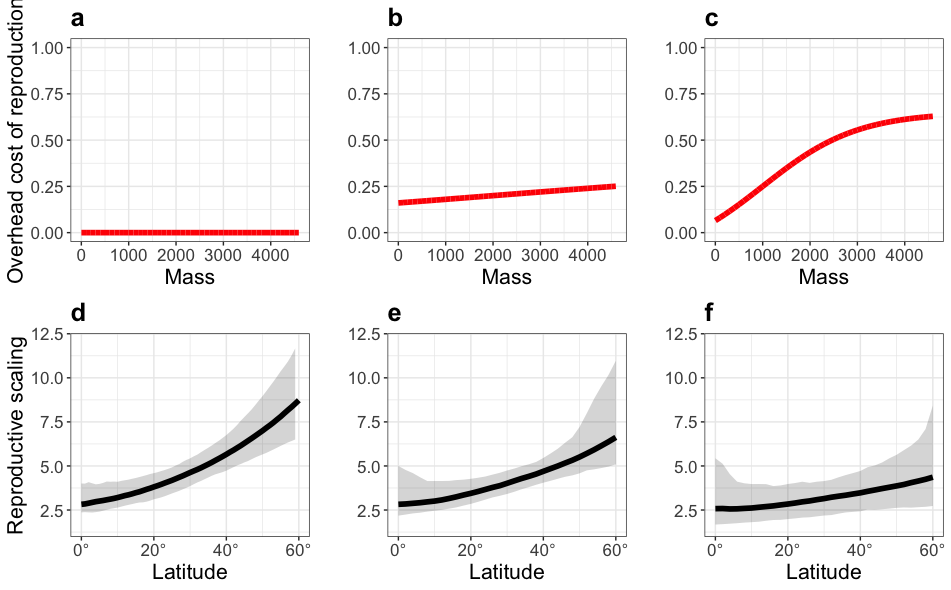

Supplement: S4 Fig — Panels (a-c) show 3 different relationships between the overhead cost of reproduction and female size. Panels (d-f) show the reproductive scaling predicted by the life history optimisation model given different reproductive costs. Panels (a) and (d) assume no overhead cost to reproduction (cr = 0). Panels (b) and (e) assume an overhead cost of reproduction that increases slightly with female size (cr=0.4/(1+e−0.002*(w−2000)), where w is female weight). Panels (c) and (f) assume a steep increase in overhead cost of reproduction with female size (cr=0.8/(1+e−0.001*(w−1000))−0.15). Predictions for reproductive scaling were generated assuming fecundity was given by: f(t)=(1−u(t))P(w)*(1−cf), where f(t) was optimised given the latitudinal gradient for M, k, and w0 (S1 Table). Because panel (a) represents the scenario where reproduction has no overhead costs, S4A Fig is identical to Fig 2A. (TIFF) [file pbio.3002114.s004.tiff]

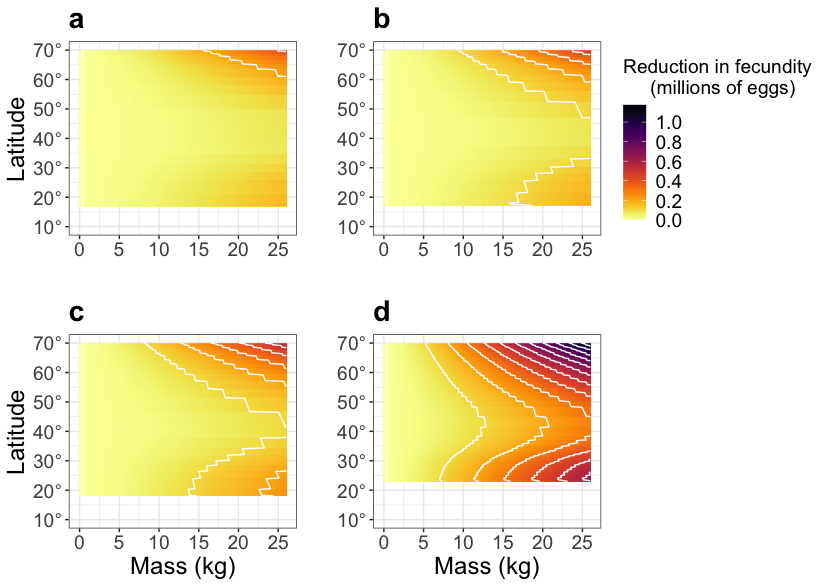

Supplement: S5 Fig — RCP2.6 and RCP8.5 scenarios are a low- and very high-emission scenario, respectively. Expected fecundity changes across latitudes for different female sizes for 2031–2050 under the RCP2.6 scenario, 0.64°C increase in SST (a), for 2081–2100 under the RCP2.6 scenario, 0.73°C increase in SST (b), for 2031–2050 under the RCP8.5 scenario, 0.95°C increase in SST (c), and for 2081–2100 under the RCP8.5 scenario, 2.58°C increase in SST (d). The colours show the reduction in the expected number of eggs, with the smallest reduction in yellow and the largest in black. The white contour lines show consecutive reductions in fecundity of 100,000 eggs. Current sea surface water temperature (SST) was assumed to be the historical mean (1981–2010) from the COBE-SST2 data provided by the NOAA/OAR/ESRL PSL (Boulder, Colorado, USA) (https://psl.noaa.gov/). (TIFF) [file pbio.3002114.s005.tiff]

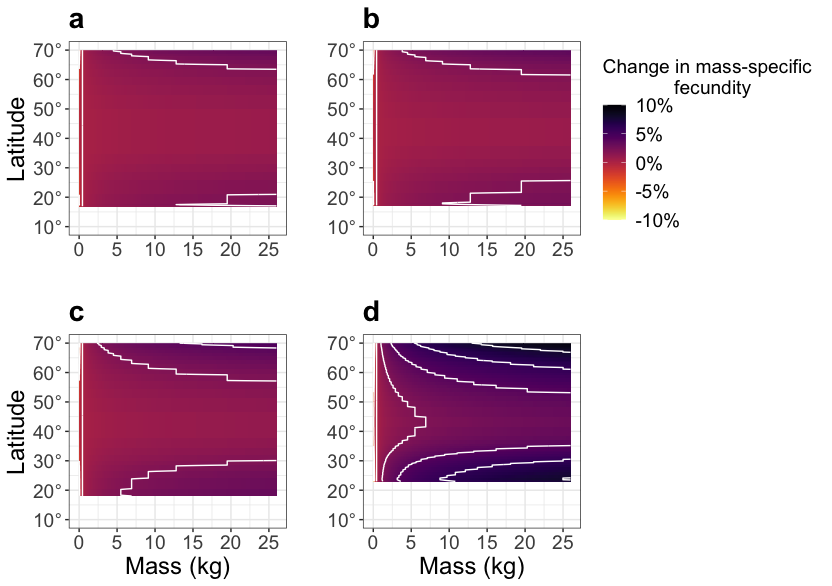

Supplement: S6 Fig — The colours show the reduction in the percentage change in mass-specific fecundity, with the largest increase in yellow and the largest decrease in black. RCP2.6 and RCP8.5 scenarios are a low- and very high-emission scenario, respectively. (a) Expected change in the mass-specific fecundity across latitudes for different female sizes for 2031–2050 under the RCP2.6 scenario (0.64°C increase in SST). (b) Expected change in the mass-specific fecundity across latitudes for different female sizes for 2081–2100 under the RCP2.6 scenario (0.73°C increase in SST). (c) Expected change in the mass-specific fecundity across latitudes for different female sizes for 2031–2050 under the RCP8.5 scenario (0.95°C increase in SST). (d) Expected change in the mass-specific fecundity across latitudes for different female sizes for 2081–2100 under the RCP8.5 scenario (2.58°C increase in SST). The colours show the change in the mass-specific fecundity, with the largest increase in percentage of mass-specific fecundity in yellow and the largest decrease in black. The white contour lines show consecutive 2% changes in mass-specific fecundity. Current sea surface water temperature (SST) was assumed to be the historical mean (1981–2010) from the COBE-SST2 data provided by the NOAA/OAR/ESRL PSL (Boulder, Colorado, USA) (https://psl.noaa.gov/). (TIFF) [file pbio.3002114.s006.tiff]

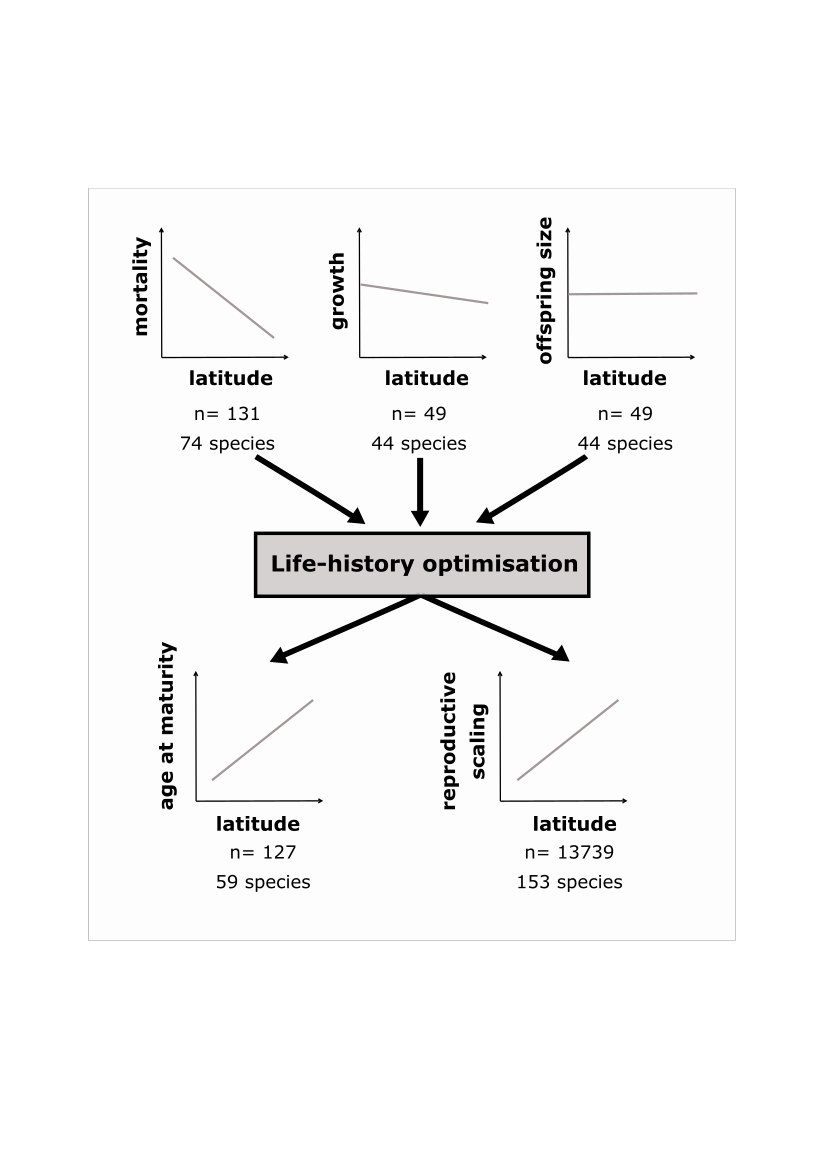

Supplement: S7 Fig — First, we compile life history data (mortality, growth, and offspring size) of marine fishes across latitudes from the literature. We used these compiled data to fit statistical models and predict life history as a function of latitude. Then, using those predictions, we found the age at maturity that would maximise lifetime reproductive output (R0) at each latitude, as well as the resulting reproductive scaling. Finally, we compared those predictions to data from the literature. (TIFF) [file pbio.3002114.s007.tiff]

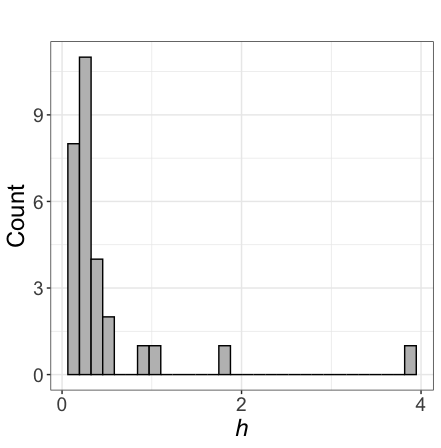

Supplement: S8 Fig — The median value equals 0.26 (first quartile is 0.18, third quartile is 0.39, mean is 0.48 ± 0.14 SE). (TIFF) [file pbio.3002114.s008.tiff]

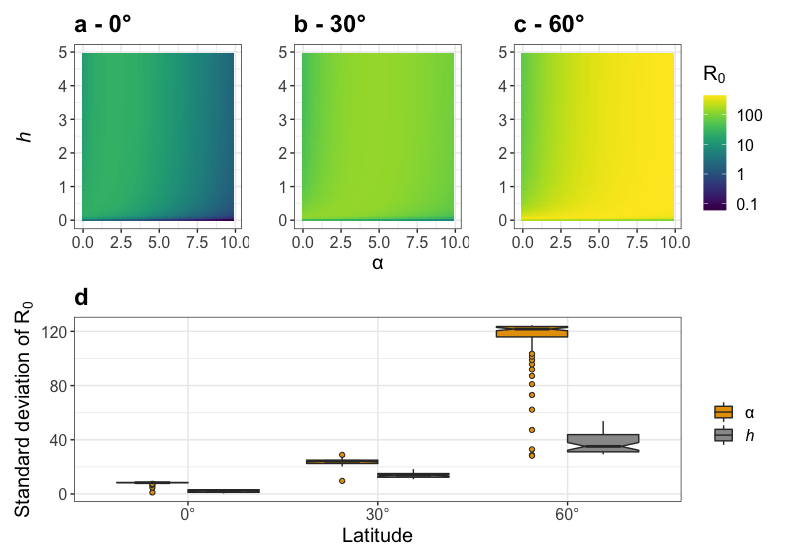

Supplement: S9 Fig — The colours show the predicted value of R0 from low (blue) to high (yellow). Panel (d) shows the distribution of standard deviations in R0 for constant h values but varying α values (labelled as α, in orange), and for constant α values but varying h values (labelled as h, in grey) at 0°, 30°, and 60° latitude. The standard deviation of R0 with variations in α ranges from 1.13 to 8.78 (median of 8.32) at 0° latitude, from 9.67 to 28.9 (median of 24.1) at 30° latitude, and from 28.21 to 124.52 (median 121.69) at 60° latitude. The standard deviation of R0 with variations in h ranges from 0.43 to 3.55 (median of 2.51) at 0° latitude, from 10.88 to 18.33 (median of 13.91) at 30° latitude, and from 29.28 to 53.80 (median 35.14) at 60° latitude. (TIFF) [file pbio.3002114.s009.tiff]

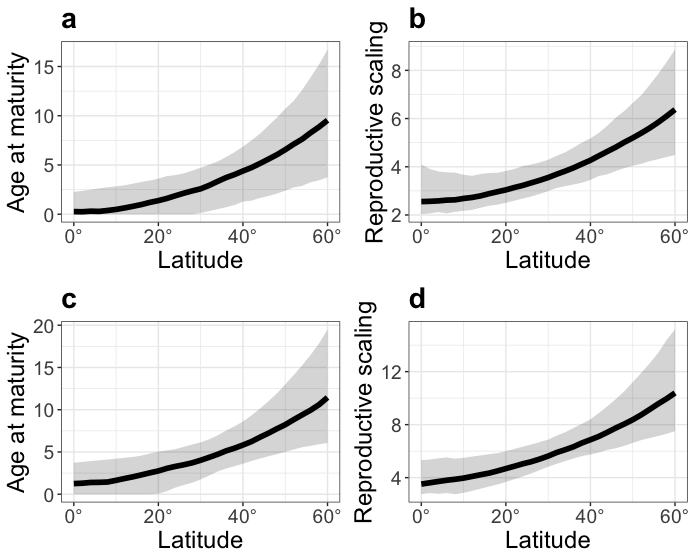

Supplement: S10 Fig — Life history predictions of the optimal reproductive schedules for the lower (h = 0.18 in panels (a) and (b)) and the upper quartile of the distribution of h values estimated from the mass at age relationship data (h = 0.39 in panels (c) and (d)). (a and c) Optimal α across latitudes. (b and d) Reproductive scaling resulting from the optimal reproductive schedule. The black lines show the medians of the posterior distributions, and the grey ribbons show the 95% credible intervals. The life history optimisation assumes mortality (M), growth (k), and offspring size (w0) change across latitudes following: Ln(D) = a+b*latitude, where D is the demographic rate. For mortality, a = −0.31 (95% CI: −0.82 to 0.20) and b = −0.02 (95% CI: −0.04 to −0.01). For growth, a = 1.47 (95% CI: 0.66 to 2.30) and b = −0.01 (95% CI: −0.03 to 0.01)). For offspring size, a = 2.67 (95% CI: 0.63 to 5.05) and b = 0.00 (95% CI: −0.05 to 0.04). All demographic estimates can be found in S1 Table. (TIFF) [file pbio.3002114.s010.tiff]

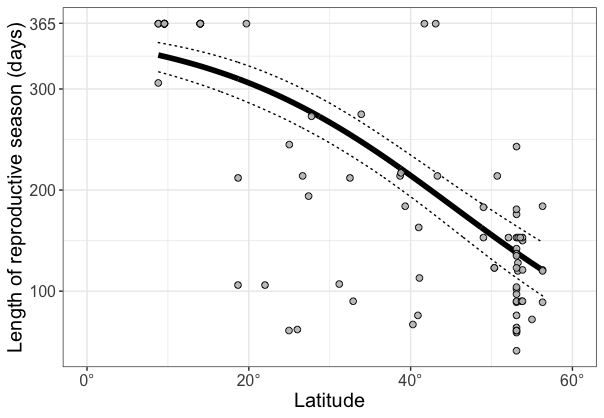

Supplement: S12 Fig — The grey points show data points corrected for phylogenetic effects. The solid lines show the model’s predictions, and the dashed lines show the 95% credible intervals. The data underlying this figure can be found in S5 Data. The model’s predictions are: P = a+b*latitude, where P is the season length (as proportion of the year), a = 2.96 (95% CI: 2.35 to 3.59) and b = −0.07 (95% CI: −0.08 to −0.05), with a logit link. (TIFF) [file pbio.3002114.s012.tiff]

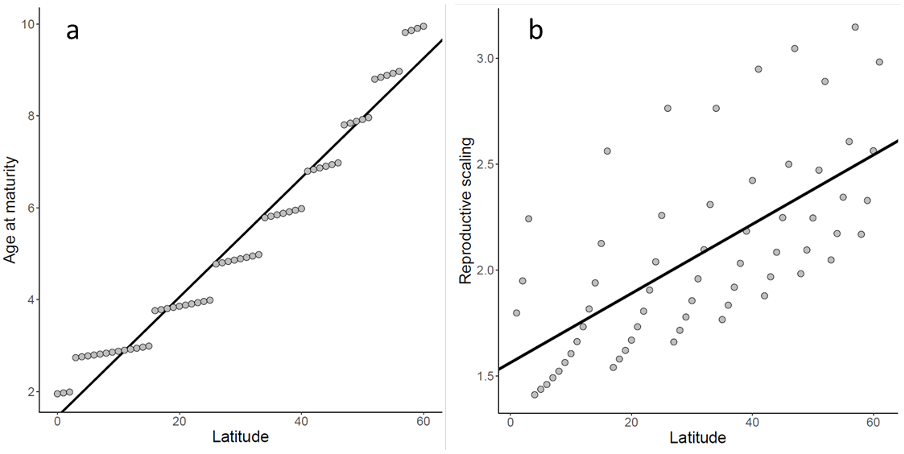

Supplement: S13 Fig — The grey points show the optimal value at each latitude. The black lines show the linear fits. (a) Optimal age at maturity (years) across latitudes. Discontinuities appear when age at maturity jumps to a next year. (b) Reproductive scaling resulting from the optimal reproductive schedule. The life history optimisation assumes mortality (M), changes across latitudes following: Ln(M) = a+b*latitude, where a = −0.31 (95% CI: −0.82 to 0.20) and b = −0.02 (95% CI: −0.04 to −0.01) (S1 Table). The model also assumes season length (P, as proportion of the year), changes across latitudes following: Ln(P) = a+b*latitude, where a = 2.96 (95% CI: 2.35 to 3.59) and b = −0.07 (95% CI: −0.08 to −0.05), with a logit link. (TIFF) [file pbio.3002114.s013.tiff]

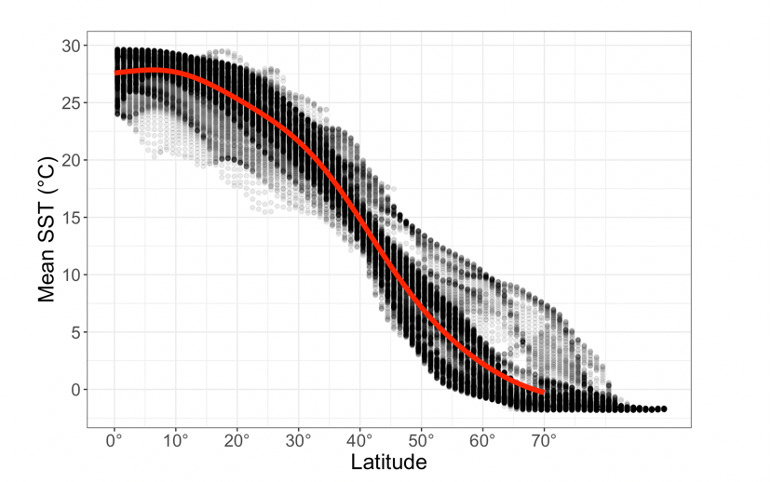

Supplement: S14 Fig — The black points show the data points, and the red line shows the model fit (from a general additive model, GAM). SST values were extracted from the COBE-SST2 data provided by the NOAA/OAR/ESRL PSL (Boulder, Colorado, USA) (https://psl.noaa.gov/). (TIFF) [file pbio.3002114.s014.tiff]

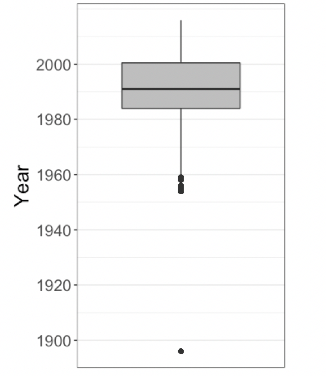

Supplement: S15 Fig — The boxplot shows the upper (2000) and lower (1984) 25% quantiles, and the horizontal black line shows the median (1991). (TIFF) [file pbio.3002114.s015.tiff]
