# Supplementary material for: Life history optimisation drives latitudinal gradients and responses to global change in marine fishes
Source: PLoS Biol. 2023 May 25;21(5):e3002114. doi: 10.1371/journal.pbio.3002114 (PMC10212075; doi:10.1371/journal.pbio.3002114)
Supplement: S1 Table — (DOCX) [file pbio.3002114.s016.docx]

| Ln(*M*) | | | |
| --- | --- | --- | --- |
|  | Estimate | Lower 95% C.I. | Upper 95% C.I. |
| Intercept | -0.31 | -0.82 | 0.20 |
| Latitude | -0.02 | -0.04 | -0.01 |
|  |  |  |  |
| Ln(*k*) | | | |
|  | Estimate | Lower 95% C.I. | Upper 95% C.I. |
| Intercept | 1.47 | 0.66 | 2.30 |
| Latitude | -0.01 | -0.03 | 0.01 |
| Phylogenetic signal | 0.19 | 0.00 | 0.65 |
|  |  |  |  |
| Ln(*w_0_*) | | | |
|  | Estimate | Lower 95% C.I. | Upper 95% C.I. |
| Intercept | 2.67 | 0.63 | 5.05 |
| Latitude | -0.00 | -0.05 | 0.04 |
| Phylogenetic signal | 0.14 | 0.00 | 0.42 |

| Ln(A_50_) | | | |
| --- | --- | --- | --- |
|  | Estimate | Lower 95% C.I. | Upper 95% C.I. |
| Intercept | -0.28 | -2.10 | 1.57 |
| Latitude | 0.03 | 0.02 | 0.04 |
| Phylogenetic signal | 0.98 | 0.95 | 0.99 |
|  |  |  |  |
| Ln(fecundity) | | | |
|  | Estimate | Upper 95% C.I. | Lower 95% C.I. |
| Intercept | 5.39 | 4.95 | 5.85 |
| Latitude | -0.02 | -0.03 | -0.02 |
| Ln(mass (g)) | 1.00 | 0.94 | 1.07 |
| Ln(Mass) **x** Latitude | 0.004 | 0.003 | 0.005 |
