# Supplementary material for: Life history optimisation drives latitudinal gradients and responses to global change in marine fishes
Source: PLoS Biol. 2023 May 25;21(5):e3002114. doi: 10.1371/journal.pbio.3002114 (PMC10212075; doi:10.1371/journal.pbio.3002114)
Supplement: S1 Supporting Information — (DOCX) [file pbio.3002114.s022.docx]

**Supplementary Information**

Life-history optimisation drives latitudinal gradients and responses to global change in marine fishes

**Size-dependent mortality**

Smaller fish tend to have higher mortality rates than larger fish, both among (1) and within species (e.g.,(2,3)). To investigate whether our results were sensitive to the assumption of size-independent mortality (except for the initial burst in mortality), we modified the model to include a negative relationship between survival and mass. To do this, we calculated the maximum mass possible at each latitude (given the range of $\alpha$ values, *h* was kept constant at 0.26, and re-scaled mass relative to this value (i.e., relative mass, $m)$. *M* at each latitude was then multiplied by a factor *d,* where $d=1-qm$, such that the larger the value of *q,* the steeper the decrease in *M* with relative size. Mortality of the largest size classes due to senescence (e.g., (3)) was ignored because the probability of surviving to those sizes is too low to qualitatively, and even substantially quantitatively, change model predictions.

Size-dependent mortality did not qualitatively change the results of the optimisation. Age at maturity ($\alpha$) increased with latitude for all size-dependent mortality relationships (S2a and S2b Fig) increasing more rapidly when mortality declined more steeply with size. Similarly, reproductive scaling increased with increasing latitude in all cases, increasing more rapidly when size-dependency of mortality was stronger (S2c Fig). These results are consistent with expectations from life-history theory (4): maturity is further delayed when mortality decreases more steeply with size.

**Overhead cost of reproduction**

The life-history optimisation model used to generate predictions in Figs 1a and 2a makes no assumptions about the proportion of energy invested into reproduction that directly translates to egg energy content. However, if the overhead costs of reproduction (e.g., energy spent in mating behaviour or in maintaining reproductive tissue, burgeoning gametes or offspring), increase with size, the expected reproductive scaling exponent would be lower. For example, the plots below show three different assumptions about the overhead costs of reproduction: 1) no overhead cost (which is yields the same reproductive scaling than an overhead cost that is independent of female size) (S4a and S4d Fig), 2) a shallow increase in the overhead cost of reproduction with female size (S4b and S4e Fig), and 3) a steep increase in the overhead cost of reproduction with size (S4c and S4f Fig). The shallowest reproductive scaling arises when the overhead costs of reproduction increase steeply with size.

**Predicted size-specific changes in reproductive output with climate change**

In the low emissions scenarios, most female sizes are not predicted to suffer a reduction in reproductive output but fish larger than ~15kg are predicted to have reduced fecundities of up to ~300 or 330 thousand eggs at the highest latitudes (mid- and late-century predictions, respectively) (S5a and S5b Fig). Predictions for mid-century fecundities under the high emission scenario are similar to predictions under the low emissions scenario but with smaller sizes suffering reductions particularly at high and low latitudes, with a maximum reduction of ~880 thousand eggs for a 25kg fish near the poles (S5c Fig). In contrast, late-century predictions for the high emissions scenario predicts that all but the smallest fish will suffer reductions in fecundity, the largest reduction in average fecundity being close to 2 million eggs in the largest polar females (S5d Fig).

**Kozłowski’s life-history model**

In Kozłowski’s life-history model (5) growth also changes throughout ontogeny as resources are diverted from growth to reproduction. In this model, every year is split into a productive season – when there is surplus energy available for growth or reproduction – and an unproductive season. The productive season is divided into time spent growing and time spent reproducing, with a maximum of one switch from growth to reproduction per season. This means that in a productive season surplus energy can be invested entirely to growth, entirely to reproduction, or initially to growth and then to reproduction. As with Day and Talyor’s model, this model assumes that production rate *P*(*w*) depends on weight (*w*) as defined by:

$P\left( w \right)=aw^{b}$,

(Eq. S1)

where *a* and *b* are constant (here fixed at *a*=0.025 and *b*=3/4). Weight before maturity can be solved from Eq. S1 (which is the general form of Eq. 4 when *t<*$\alpha$*,* with *b* replacing 3/4):

$$w_{t}=\left[ (k\left( 1-b \right)t+{w_{0}}^{1-b} \right]^{\frac{1}{1-b}}.$$

(Eq. S2)

After maturity, weight before switching to reproductive allocation depends on the resources obtained before switching:

$$w_{t+1}=\left[ (k\left( 1-b \right)(T_{s}-E_{t+1})+{w_{t}}^{1-b} \right]^{\frac{1}{1-b}}.$$

(Eq. S3)

$T_{s}$ is the length of the productive season in days and $E_{t+1}$ is the number of days in which surplus energy is allocated into reproduction in year *t+1*. Age at maturity corresponds to the first day in which energy was invested into reproduction (in unit of years). Energy invested into reproduction is defined as:

$f_{t+1}=P\left( w_{t+1} \right)E_{t+1}$.

(Eq. S4)

Life-time reproductive success was calculated as a sum of reproductive allocation in consecutive years weighted by a probability of surviving to spawning taking place at the beginning of the next productive season.

In this model, $E$ was optimised at each time point to maximise life-time reproductive success (equivalent to maximization of $R_{0}$) given a latitude-dependent mortality, with hjkb R procedure (6). In Kozłowski’s (1996) model, resources gathered for reproduction were released as eggs at the end of the productive season. Here we assumed more realistically that these resources are released at spawning taking place at the beginning of the next productive season.

Examples of optimal trajectories of growth according to Kozłowski’s model under different mortality rates are presented in S11 Fig as grey circles. Solid lines in this figure represent growth trajectories fitted with Day and Taylor’s model (Eq. 3), assuming the same initial size *w*_0_, growth rate parameter *k* and age at maturity. Fit is almost ideal for middle mortality, and good for both low and high mortality. This similarity justifies applying simpler Day and Taylor’s model in the paper.

We used the same estimates of *M* across latitudes than for Day and Taylor’s model. Data on the length of the productive season (*T_s_*) was very scarce, hence we used length of the reproductive season as a proxy. We expect the duration of the reproductive season to be proportional to the length of the productive season. Typically, the length of the reproductive season was specified at a resolution of months and not days, so we use these estimates only as a coarse guide of how reproductive season changes across latitudes. We compiled a total of 76 data points for 58 species from 43 references. Latitudes ranged from 8.8 to 56.3°.

To predict *T_s_*, we divided the number of days of reproductive season by 365 to get the proportion of the year in which individuals were reproducing. Whenever this proportion was one, we set it to 0.999 in order to assume a beta distribution, which is appropriate for proportional data but that excludes 1 and 0 values. The reproductive proportion of the year was predicted with a linear mixed effect model with absolute latitude as a fixed effect.

The reproductive season encompassed a higher proportion of the year in the tropics than in the poles (0.95 at 0° and 0.28 at 60°) (R^2^= 0.53, C.I.: 0.43-0.59) (S12 Fig). Using these estimates of *T_s_* and the estimates of *M* across latitudes presented in the main text, there was a positive relationship between optimal age at maturity and latitude (S13a Fig). Reproductive scaling increased monotonically with latitude only for latitudes where optimal age at maturity fell within the same year (S13b Fig), but there is general trend for increase of this scaling with latitude (black line in S13b Fig).

1. Charnov EL, Gillooly JF. Size and temperature in the evolution of fish life histories. Integr Comp Biol. 2004;44(6):494–7.

2. Larsen L-H, Pedersen T. Migration, growth and mortality of released reared and wild cod (Gadus morhua L.) in Malangen, northern Norway. Sarsia. 2002;87(2):97–109.

3. Hampton J. Natural mortality rates in tropical tunas: size really does matter. Can J Fish Aquat Sci. 2000;57(5):1002–10.

4. Kozłowski J, Wiegert RG. Optimal allocation of energy to growth and reproduction . Vol. 29, Theoretical Population Biology . United States : Elsevier Inc ; 1986. p. 16–37.

5. Kozłowski J. Optimal Allocation of Resources Explains Interspecific Life-History Patterns in Animals with Indeterminate Growth. Proc R Soc B Biol Sci. 1996;263(1370):559–66.

6. Varadhan R, Borchers HW, Bechard V. dfoptim: Derivative-Free Optimization. 2020.
