# Supplementary material for: Life history optimisation drives latitudinal gradients and responses to global change in marine fishes
Source: PLoS Biol. 2023 May 25;21(5):e3002114. doi: 10.1371/journal.pbio.3002114 (PMC10212075; doi:10.1371/journal.pbio.3002114)
Supplement: S1 Compiled Data References — (PDF) [file pbio.3002114.s023.pdf]

Mortality references

Aasen O (1963) Length and growth of the porbeagle (Lamna nasus, Bonnaterre) in the North West Atlantic.

Adam MS, Sibert J, Itano D, Holland K (2003) Dynamics of bigeye (Thunnus obesus) and yellowfin (T. albacares) tuna in Hawaii's pelagic fisheries: analysis of tagging data with a bulk transfer model incorporating size-specific attrition.

Archibald CP, Shaw W, Leaman BM (1981) Growth and mortality estimates of rockfishes (Scorpaenidae) from B.C. coastal waters, 1977-1979.

Bakken E (1987) Growth, biomass, and production of a small unexploited plaice stock in St. Margaret's Bay, Nova Scotia.

Banerji SK (1973) An assessment of the exploited pelagic fisheries of the Indian seas. 114–136

Beyer JE, Kirchner CH, Holtzhausen JA (1999) A method to determine size-specific natural mortality applied to westcoast steenbras (Lithognathus aureti) in Namibia . Fish Res 41:133–153

Block BA, Whitlock R, Schallert RJ, Wilson S, Stokesbury MJW, Castleton M, Boustany A (2019) Estimating Natural Mortality of Atlantic Bluefin Tuna Using Acoustic Telemetry . Sci reports 9:4914–4918

Chen D-G, Xiao Y (2006) A general model for analyzing data from mark-recapture experiments with an application to the Pacific halibut. Environ Ecol Stat 13:149–161

Choat JH, Robertson DR, Ackerman JL, Posada JM (2003) An age-based demographic analysis of the Caribbean stoplight parrotfish Sparisoma viride. Mar Ecol Prog Ser 246:265–277

Cowen L, Walsh SJ, Schwarz CJ, Cadigan N, Morgan J (2009) Estimating exploitation rates of migrating yellowtail flounder (Limanda ferruginea) using multistate mark–recapture methods incorporating tag loss and variable reporting rates. Can J Fish Aquat Sci 66:1245–1255

Curtis JMR, Vincent ACJ (2006) Life history of an unusual marine fish: survival, growth and movement patterns of Hippocampus guttulatus Cuvier 1829. J Fish Biol 68:707–733

Fletcher WJ (1995) Application of the otolith weight–age relationship for the pilchard, Sardinops sagax neopilchardus. Can J Fish Aquat Sci 52:657–664

Garrod DJ (1967) Population dynamics of the Arcto-Norwegian cod. J Fish Board Canada 24:145–190

Gjösæter J (1973) Age, growth, and mortality of the mygtophid fish, Benthosema glaciale (Reinhardt), from Western Norway. Sarsia 52:1–14

Le Guen J-C (1971) Dynamique des populations de Pseudotolithus (Fonticulus) elongatus (Bowd. 1825) poissons Sciaenidae.

Gust N, Choat J, Ackerman J (2002) Demographic plasticity in tropical reef fishes. Mar Biol 140:1039–1051

Hampton J (2000) Natural mortality rates in tropical tunas: size really does matter. Can J Fish Aquat Sci 57:1002–1010

den Heyer CE, Schwarz CJ, Trzcinski MK (2013) Fishing and natural mortality rates of Atlantic halibut estimated from multiyear tagging and life history. Trans Am Fish Soc 142:690–702

Horn PL (1993) Growth, age structure, and productivity of jack mackerels (Trachurus spp.) in New Zealand waters. New Zeal J Mar Freshw Res 27:145–155

Hutchings K, Griffiths MH (2010) Life-history strategies of Umbrina robinsoni (Sciaenidae) in warm-temperate and subtropical South African marine reserves . African J Mar Sci 32:37–53

Iversen SA, Zhu D, Johannessen A, Toresen R (1993) Stock size, distribution and biology of anchovy in the Yellow Sea and East China Sea. Fish Res 16:147–163
Kovacic M (2006) Age structure, growth and mortality of the striped goby, Gobius vittatus (Gobiidae) in the northern Adriatic Sea. Sci Mar 70:635–641
Krishnamoorthi B (1976) A note on mortality rates and yield per recruit in Nemipterus japonicus (Block). Indian J Fish 23:252–255
Larsen L-H, Pedersen T (2002) Migration, growth and mortality of released reared and wild cod (Gadus morhua L.) in Malangen, northern Norway. Sarsia 87:97–109
Leaman BM, Nagtegaal DA (1987) Age Validation and Revised Natural Mortality Rate for Yellowtail Rockfish . Trans Am Fish Soc 116:171–175
Linkowski TB (1985) Population biology of the myctophid fish Gymnoscopelus nicholsi (Gillbert, 1911) from the western South Atlantic. J Fish Biol 27:683–698
Lorance P, Dupouy H, Allain V (2001) Assessment of the roundnose grenadier (Coryphaenoides rupestris) stock in the Rockall Trough and neighbouring areas (ICES Sub-areas V–VII). Fish Res 51:151–163
MacCall AD (1973) The mortality rate of Engraulis mordax in southern California. Calif Coop Ocean Fish Investig
Macpherson E, García-Rubies A, Gordoa A (2000) Direct estimation of natural mortality rates for littoral marine fishes using populational data from a marine reserve. Mar Biol 137:1067–1076
Mathews CP (1975) Some observations on the ecology and the population dynamics of Merluccius angustimanus in the South Gulf ofCalifornia. J Fish Biol 7:83–94
Mitani F, Shojima E (1966) Studies on the resources of the jack mackerel, Trachurus japonicus (Temminck et Schlegel), in the East China Sea-III,-Natural and fishing mortality coefficients. Bull Japanese Soc Sci Fish 32:57–63
Moses BS (1988) Growth, mortality and potential yield of bonga, Ethmalosa fimbriata (Bowdich 1825) of Nigerian inshore waters. Fish Res 6:233–247
Newman SJ, Cappo M, Williams DM (2000a) Age, growth, mortality rates and corresponding yield estimates using otoliths of the tropical red snappers, Lutjanus erythropterus, L. malabaricus and L. sebae, from the central Great Barrier Reef. Fish Res 48:1–14
Newman SJ, Cappo M, Williams DM (2000b) Age, growth and mortality of the stripey, Lutjanus carponotatus (Richardson) and the brown-stripe snapper, L. vitta (Quoy and Gaimard) from the central Great Barrier Reef, Australia . Fish Res 48:263–275
O’Connell V, Brylinsky C, Carlile D (2002) Demersal shelf rockfish stock assessment for 2003. Alaska Dep Fish Game IJ02-44
Parrish RH, MacCall AD (1978) Climatic variation and exploitation in the Pacific mackerel fishery. State of California, Resources Agency, Department of Fish and Game,
Pauly D (1980) On the interrelationships between natural mortality, growth parameters, and mean environmental temperature in 175 fish stocks. ICES J Mar Sci 39:175–192
Pinhorn AT (1975) Estimates of natural mortality for the cod stock complex in ICNAF Divisions 2J, 3K and 3L.
Potts WM, Sauer WHH, Childs A-R, Duarte ADC (2008) Using baseline biological and ecological information to design a Traffic Light Precautionary Management Framework for leerfish Lichia amia (Linnaeus 1758) in southern Angola . African J Mar Sci 30:113–121
Rudershausen PJ, Williams EH, Buckel JA, Potts JC, Manooch III CS (2008) Comparison of reef fish catch per unit effort and total mortality between the 1970s and 2005-2006 in Onslow Bay, North Carolina. Trans Am Fish Soc 137:1389–1405
Russ GR, Lou DC, Higgs JB, Ferreira BP (1998) Mortality rate of a cohort of the coral trout,

Plectropomus leopardus, in zones of the Great Barrier Reef Marine Park closed to fishing. Mar Freshw Res 49:507–511
Shlibanov VI (1989) Growth and natural mortality of Patagonian rockcod (Patagonothothen guntheri shagensis) from Shag Rocks shelf. Sci Comm Conserv Antarct Mar Living Resour Sel Sci Pap 111–121
Siddeek MSM (1989) The estimation of natural mortality in Irish Sea plaice, Pleuronectes platessa L., using tagging methods. J Fish Biol 35:145–154
Sinclair AF (2001) Natural mortality of cod (Gadus morhua) in the Southern Gulf of St Lawrence. ICES J Mar Sci 58:1–10
Stevens JD, Hausfeld HF (1982) Age determination and mortality estimates on an unexploited population of Jack mackerel Trachurus declivis (Jenyns, 1841) from south-east Australia. Commonwealth Scientific and Industrial Research Organization, Marine …,
Sudjastani T (1974) Species of Rastrelliger in the Java Sea, their taxonomy, morphometry and population dynamics.
Svedäng H (1999) Vital population statistics of the exploited eel stock on the Swedish west coast. Fish Res 40:251–265
Tanaka E (2006) Simultaneous estimation of instantaneous mortality coefficients and rate of effective survivors to number of released fish using multiple sets of tagging experiments. Fish Sci 72:710–718
Tanasichuk RW (2000) Age-specific natural mortality rates of adult Pacific herring (Clupea pallasi) from southern British Columbia. Can J Fish Aquat Sci 57:2258–2266
Thomas JC (1968) Management of the white seabass (Cynoscion nobilis) in California waters. Calif Dep Fish Game, Fish Bull 142:
Vooren CM (1977) Growth and mortality of tarakihi (Pisces: Cheilodactylidae) in lightly exploited populations. New Zeal J Mar Freshw Res 11:1–22
Wakefield CB, Moran MJ, Tapp NE, Jackson G (2007) Catchability and selectivity of juvenile snapper (Pagrus auratus, Sparidae) and western butterfish (Pentapodus vitta, Nemipteridae) from prawn trawling in a large marine embayment in Western Australia . Fish Res 85:37–48
Wang Y (2016) Changes in natural mortality of Atlantic cod (Gadus morhua) on Eastern Georges Bank. J Ocean Univ China 15:879–889
Wang Y, Liu Q (2006) Estimation of natural mortality using statistical analysis of fisheries catch-at-age data . Fish Res 78:342–351
Wilderbuer TK, Turnock BJ (2009) Sex-specific natural mortality of arrowtooth flounder in Alaska: Implications of a skewed sex ratio on exploitation and management. North Am J Fish Manag 29:306–322
Williams AJ, Davies CR, Mapstone BD, Currey LM, Welch DJ, Begg GA, Ballagh AC, Choat JH, Murchie CD, Simpfendorfer CA (2009) Age-based demography of humpback grouper Cromileptes altivelis: implications for fisheries management and conservation. Endanger Species Res 9:67–79
Williams AJ, Mapstone BD, Davies CR (2007) Spatial patterns in cohort-specific mortality of red throat emperor, Lethrinus miniatus, on the Great Barrier Reef . Fish Res 84:328–337
Winters GH (1983) Analysis of the biological and demographic parameters of northern sand lance, Ammodytes dubius, from the Newfoundland Grand Bank. Can J Fish Aquat Sci 40:409–419
Young PH (1963) The Kelp Brass (Paralabrax Clathratus) and Its Fishery, 1947-1958. Department of Fish and Game, the Resources Agency, State of California,

Growth and offspring size references

Akita Y, Tachihara K (2014) Age, growth, maturity, and sex changes of monogrammed monocle bream Scolopsis monogramma in the waters around Okinawa-jima Island, Japan. Fish Sci 80:679–685

Alcazar Alvarez JL, Carrasco Fidalgo JF, Llera Gonzalez EM, Menendez de la Hoz M, Ortea Rato JA (1983) Biologia, dinamica y pesca de la merluza en Asturias.

Carvalho N, Perrotta RG, Isidro E (2002) Age, growth and maturity in the chub mackerel (Scomber japonicus Houttuyn, 1782) from the Azores.

Cengiz Ö (2012) Age, growth, mortality and reproduction of the chub mackerel (Scomber japonicus Houttuyn, 1782) from Saros Bay (Northern Aegean Sea, Turkey). Turkish J Fish Aquat Sci 12:

Cergole MC, Rossi-Wongtschowski CLDB (2005) Análise das principais pescarias comerciais da região Sudeste-Sul do Brasil: dinâmica populacional das espécies em explotação. Instituto Oceanográfico,

Ceyhan T, Akyol O, Ayaz A, Juanes F (2007) Age, growth, and reproductive season of bluefish (Pomatomus saltatrix) in the Marmara region, Turkey. ICES J Mar Sci 64:531–536

Daley T (2018) Growth and reproduction of Atlantic chub mackerel (Scomber colias) in the Northwest Atlantic.

Georgina Gluyas-Millan M, Quinonez-Velazquez C (1997) Age, Growth, and Reproduction of Pacific Mackerel Scomber Japonicus in the Gulf of California . Bull Mar Sci 61:837–847

Grandcourt EM, Al Abdessalaam TZ, Francis F, Al Shamsi AT (2005) Preliminary assessment of the biology and fishery for the narrow-barred Spanish mackerel, Scomberomorus commerson (Lacépède, 1800), in the southern Arabian Gulf. Fish Res 76:277–290

Hutchings K, Griffiths MH (2010) Life-history strategies of Umbrina robinsoni (Sciaenidae) in warm-temperate and subtropical South African marine reserves . African J Mar Sci 32:37–53

Hwang S-D, Kim J-Y, Lee T-W (2008) Age, Growth, and Maturity of Chub Mackerel off Korea . North Am J Fish Manag 28:1414–1425

Hyndes GA, Potter IC (1996) Comparisons between the age structures, growth and reproductive biology of two co-occurring sillaginids, Sillago robusta and S. bassensis, in temperate coastal waters of Australia. J Fish Biol 49:14–32

Llompart FM, Colautti DC, Maiztegui T, Cruz-Jiménez AM, Baigún CRM (2013) Biological traits and growth patterns of pejerrey Odontesthes argentinensis. J Fish Biol 82:458–474

Love MS, Westphal W V (1981) Growth, reproduction, and food habits of olive rockfish Sebastes serranoides, off central California . Fish Bull 79:533–545

Magnussen E (2007) Interpopulation comparison of growth patterns of 14 fish species on Faroe Bank: are all fishes on the bank fast-growing? J Fish Biol 71:453–475

Mathews CP (1975) Some observations on the ecology and the population dynamics of Merluccius angustimanus in the South Gulf ofCalifornia. J Fish Biol 7:83–94

Murty VS (1984) Observations on the fisheries of threadfin breams (Nemipteridae) and on the biology of Nemipterus laponicus (block) from Kakinada. Indian J Fish 31:1–18

Murua H (2003) Population structure, growth and reproduction of roughhead grenadier on the Flemish Cap and Flemish Pass. J Fish Biol 63:356–373

Pember MB, Newman SJ, Hesp SA, Young GC, Skepper CL, Hall NG, Potter IC (2005) Biological parameters for managing the fisheries for blue and king threadfin salmons, estuary rockcod, Malabar grouper and mangrove jack in north-western Australia.

Potts WM, Sauer WHH, Childs A-R, Duarte ADC (2008) Using baseline biological and ecological information to design a Traffic Light Precautionary Management Framework for leerfish Lichia amia (Linnaeus 1758) in southern Angola . African J Mar Sci 30:113–121

Ross JL, Stevens TM, Vaughan DS (1995) Age, growth, mortality, and reproductive biology of red drums in North Carolina waters. Trans Am Fish Soc 124:37–54

Dos S. Lewis D, Fontoura NF (2005) Maturity and growth of Paralonchurus brasiliensis females in southern Brazil (Teleostei, Perciformes, Sciaenidae). J Appl Ichthyol 21:94–100

Sainsbury KJ, Whitelaw AW (1984) Biology of Peron's threadfin bream, Nemipterus peronii (Valenciennes), from the North West Shelf of Australia. Mar Freshw Res 35:167–185

Salerno DJ, Burnett J, Ibara RM (2001) Age, growth, maturity, and spatial distribution of bluefish, Pomatomus saltatrix (Linnaeus), off the northeast coast of the United States, 1985-96. J Northwest Atl Fish Sci 29:31–40

Sousa MI, Gislason H (1985) Reproduction, age and growth of the Indian mackerel, Rastrelliger kanagurta (Cuvier, 1816) from Sofala Bank, Mozambique. Rev Investig Pesqueira 14:1–28

Stevens D, Smith MH, Grimes P, Devine J, Sutton C, MacGibbon D, Maolagáin CÓ (2010) Age, growth, and maturity of four New Zealand rattail species. NZ Aquat Environ Biodiv Rept 39

Wakefield CB, Newman SJ, Molony BW (2010) Age-based demography and reproduction of hapuku, Polyprion oxygeneios, from the south coast of Western Australia: implications for management. ICES J Mar Sci 67:1164–1174

Age at maturity references

Abdussamad EM, Mohamad Kasim H, Achayya P (2006) Fishery and population characteristics of Indian mackerel, Rastrelliger kanagurta (Cuvier) at Kakinada. Indian J Fish 53:77–83

Abdussamad EM, Pillai NGK, Mohamad Kasim H, Mohamed O (2010) Fishery, biology and population characteristics of the Indian mackerel, Rastrelliger kanagurta (Cuvier) exploited along the Tuticorin coast. Indian J Fish 57:17–21

Ajiad A, Jakobsen T, Nakken O (1999) Sexual difference in maturation of Northeast Arctic cod. J Northwest Atl Fish Sci 25:

Akita Y, Tachihara K (2014) Age, growth, maturity, and sex changes of monogrammed monocle bream Scolopsis monogramma in the waters around Okinawa-jima Island, Japan. Fish Sci 80:679–685

Alcazar Alvarez JL, Carrasco Fidalgo JF, Llera Gonzalez EM, Menendez de la Hoz M, Ortea Rato JA (1983) Biologia, dinamica y pesca de la merluza en Asturias.

Bowering WR (1983) Age, growth, and sexual maturity of Greenland halibut, Reinhardtius hippoglossoides (Walbaum). Fish Bull 81:599

Brodziak J, Mikus R (2000) Variation in life history parameters of Dover sole, Microstomus pacificus, off the coasts of Washington, Oregon, and northern California. Fish Bull 98:661

Carvalho N, Perrotta RG, Isidro E (2002) Age, growth and maturity in the chub mackerel (Scomber japonicus Houttuyn, 1782) from the Azores.

Cengiz Ö (2012) Age, growth, mortality and reproduction of the chub mackerel (Scomber japonicus Houttuyn, 1782) from Saros Bay (Northern Aegean Sea, Turkey). Turkish J Fish Aquat Sci 12:

Claereboudt MR, McIlwain JL, Al-Oufi HS, Ambu-Ali AA (2005) Patterns of reproduction and spawning of the kingfish (Scomberomorus commerson, Lacepede) in the coastal waters of the Sultanate of Oman. Fish Res 73:273–282

Corriero A, Karakulak S, Santamaria N, Deflorio M, Spedicato D, Addis P, Desantis S, Cirillo F, Fenech-Farrugia A, Vassallo-Agius R (2005) Size and age at sexual maturity of female bluefin tuna (Thunnus thynnus L. 1758) from the Mediterranean Sea. J Appl Ichthyol 21:483–486

Cubillos L, Alarcón R, Bucarey D, Canales M, Sobarzo P, Vilugrón L, Hernández A, Sepúlveda A, Véjar F (1998) Evaluación indirecta del stock de anchoveta y sardina común en la zona centro-sur. Inf Técnicos FIP, FIP-IT/96-10 223:

Daley T (2018) Growth and reproduction of Atlantic chub mackerel (Scomber colias) in the Northwest Atlantic.

Delgado J, Reis S, González JA, Isidro E, Biscoito M, Freitas M, Tuset VM (2013) Reproduction and growth of A phanopus carbo and A. intermedius (T eleostei: T richiuridae) in the northeastern A tlantic. J Appl Ichthyol 29:1008–1014

Dorval E, McDaniel JD, Macewicz BJ, Porzio DL (2015) Changes in growth and maturation parameters of Pacific sardine Sardinops sagax collected off California during a period of stock recovery from 1994 to 2010. J Fish Biol 87:286–310

Echeverria TW (1987) Thirty-four species of California rockfishes: Maturity and seasonality of reproduction. Fish Bull 85:229–250

Farley JH, Clear NP, Leroy B, Davis TLO, McPherson G (2006) Age, growth and preliminary estimates of maturity of bigeye tuna, Thunnus obesus, in the Australian region. Mar Freshw Res 57:713–724

Gluyas-Millan MG, Quinonez-Velazquez C (1997) Age, Growth, and Reproduction of Pacific Mackerel Scomber Japonicus in the Gulf of California. Bull Mar Sci 61:837–847

Grandcourt EM, Al Abdessalaam TZ, Francis F, Al Shamsi AT (2005) Preliminary assessment of the biology and fishery for the narrow-barred Spanish mackerel, Scomberomorus commerson (Lacépède, 1800), in the southern Arabian Gulf. Fish Res 76:277–290

Hourigan TF, Radtke RL (1989) Reproduction of the Antarctic fish Nototheniops nudifrons. Mar Biol 100:277–283

Hwang S-D, Kim J-Y, Lee T-W (2008) Age, Growth, and Maturity of Chub Mackerel off Korea . North Am J Fish Manag 28:1414–1425

Joshi KK (2005) Biology and population dynamics of Nemipterus mesoprion (Bleeker) off Cochin. Indian J Fish 52:315–322

Love MS, Westphal W V (1981) Growth, reproduction, and food habits of olive rockfish Sebastes serranoides, off central California . Fish Bull 79:533–545

Magnussen E (2007) Interpopulation comparison of growth patterns of 14 fish species on Faroe Bank: are all fishes on the bank fast-growing? J Fish Biol 71:453–475

Minto C, Nolan CP (2006) Fecundity and maturity of orange roughy (Hoplostethus atlanticus Collett 1889) on the Porcupine Bank, Northeast Atlantic. Environ Biol Fishes 77:39–50

O'Brien L, Burnett J, Mayo RK (1993) Maturation of nineteen species of finfish off the northeast coast of the United States, 1985-1990.

Pitt TK (1975) Changes in abundance and certain biological characteristics of Grand Bank American plaice, Hippoglossoides platessoides. J Fish Board Canada 32:1383–1398

Salerno DJ, Burnett J, Ibara RM (2001) Age, growth, maturity, and spatial distribution of bluefish, Pomatomus saltatrix (Linnaeus), off the northeast coast of the United States, 1985-96. J Northwest Atl Fish Sci 29:31–40

Stanton Hales L (1987) Distribution, abundance, reproduction, food habits, age, and growth of round scad, Decapterus punctatus, in the South Atlantic Bight. Fish Bull 85:

Vasconcelos J, Afonso-Dias M, Faria G (2012) Atlantic chub mackerel (Scomber colias) spawning season, size and age at first maturity in Madeira waters. Arquipelago Life Mar Sci 43–51

Wassef E, El Emary H (1989) Contribution to the biology of bass, Dicentrarchus labrax L. in the Egyptian Mediterranean waters off Alexandria. Cybium (Paris) 13:327–345

Wu C-C, Weng J-S, Liu K-M, Su W-C (2008) Reproductive biology of the notchedfin threadfin bream, Nemipterus peronii (Nemipteridae), in waters of southwestern Taiwan. Zool Stud 47:103

Yamaguti N (1967) Desova da pescada-foguete, Macrodon ancylodon. Bol do Inst Ocean 16:101–106

Fecundity references

Abdullah AHJ, AI-Noor SS (2015) Observations on some reproductive features of Carasobarbus luteus (Heckel, 1843) from the Shatt Al-Arab River, Southern Iraq. Mesopotamian J Mar Sci 30:142–151

Adebiyi FA (2013) The sex ratio, gonadosomatic index, stages of gonadal development and fecundity of Sompat grunt, Pomadasys jubelini (Cuvier, 1830). Pak J Zool 45:

Al-Ogaily SM, Hussain A (1990) Biology of grunt Plectorhynchus pictus (Thunberg) 1972,(Haemulidae, Teleostei, Percoidei) from the Red Sea (Jizan area). Fish Res 9:119–130

Alonso-Fernández A, Vallejo AC, Saborido-Rey F, Murua H, Trippel EA (2009) Fecundity estimation of Atlantic cod (Gadus morhua) and haddock (Melanogrammus aeglefinus) of Georges Bank: Application of the autodiametric method . Fish Res 99:47–54

Alvarez B (2008) No Title. Instituto Politecnico Nacional

Amenzoui K, Ferhan-Tachinante F, Yahyaoui A, Kifani S, Mesfioui AH (2006) Analysis of the cycle of reproduction of Sardina pilchardus (Walbaum, 1792) off the Moroccan Atlantic coast. C R Biol 329:892–901

Andreu B (1966) Fecundidad del espadín atlántico (Sprattus sprattus) del NO de España.

Azeredo F (2014) No Title. Universidade Federal do Paraná

Bagenal TB (1957) The breeding and fecundity of the long rough dab Hippoglossoides platessoides (Fabr.) and the associated cycle in condition. J Mar Biol Assoc United Kingdom 36:339–375

Balan V (1965) The fecundity and sex composition of Sardinella longiceps Val. along the Cochin coast. Indian J Fish 12:473–491

Barneche DR, Robertson DR, White CR, Marshall DJ (2018) Fish reproductive-energy output increases disproportionately with body size. Science (80-) 360:642–645

Batts BS (1972) Sexual Maturity, Fecundity, and Sex Ratios of the Skipjack Tuna, Katsuwonus pelamis (Linnaeus), in North Carolina Waters . Trans Am Fish Soc 101:626–637

Benet DL, Dick EJ, Pearson DE (2009) Life history aspects of greenspotted rockfish (Sebastes chlorostictus) from central California.

Berglund A, Rosenqvist G, Svensson I (1986) Mate choice, fecundity and sexual dimorphism in two pipefish species (Syngnathidae). Behav Ecol Sociobiol 19:301–307

Beyer SG, Sogard SM, Harvey CJ, Field JC (2015) Variability in rockfish (Sebastes spp.) fecundity: species contrasts, maternal size effects, and spatial differences. Environ Biol Fishes 98:81–100

Bleil M, Oeberst R (2005) The potential fecundity of cod in the Baltic Sea from 1993 to 1999 . J Appl Ichthyol 21:19–27

Bobko SJ, Berkeley SA (2004) Maturity, ovarian cycle, fecundity, and age-specific parturition of black rockfish . Fish Bull 102:418

Boehlert GW, Barss WH, Lamberson PB (1982) Fecundity of the widow rockfish, Sebastes entomelas, off the coast of Oregon. Fish Bull United States, Natl Mar Fish Serv

Bouain A, Siau Y (1983) Observations on the female reproductive cycle and fecundity of three species of groupers (Epinephelus) from the southeast Tunisian seashores. Mar Biol 73:211–220

Brown-Peterson NJ, Leaf RT, Schueller AM, Andres MJ (2017) Reproductive dynamics of gulf menhaden (Brevoortia patronus) in the northern gulf of Mexico: effects on stock assessments.

Buckley LJ, Smigielski AS, Halavik TA, Caldarone EM, Burns BR, Laurence GC (1991) Winter flounder Pseudopleuronectes americanus reproductive success. II. Effects of

spawning time and female size on size, composition and viability of eggs and larvae. Mar Ecol Prog Ser Oldend 74:125–135
Butcher A, Brown I (1995) Age-structure, growth and reproduction of stout whiting Sillago robusta and Japanese market trials.
Caballero-Chávez V (2011) Reproducción y fecundidad del robalo blanco (C. undecimalis) en el suroeste de Campeche. Cienc Pesq 19:35–45
Carter AB, Davies CR, Mapstone BD, Russ GR, Tobin AJ, Williams AJ (2014) Effects of region, demography, and protection from fishing on batch fecundity of common coral trout (Plectropomus leopardus) . Coral Reefs 33:751–763
Carter J, Marrow GJ, Pryor V (1994) Aspects of the ecology and reproduction of Nassau grouper (Epinephelus striatus) off the coast of Belize, Central America.
Casavola N, Rizzi E, Marano C (1996) First data on batch fecundity and relative fecundity of Sardina pilchardus (Walbaum 1792)(Clupeidae in the south-western Adriatic Sea. Boletín-Instituto Español Oceanogr 12:53–64
Cayré P (1981) Maturité sexuelle, fécondité et sex-ratio du Listao (Katsuwonus pelamis l.) des côtes d'Afrique de l'Ouest (20° N-0° N) étudiés à partir des débarquements thoniers (1977 à 1979) au port de Dakar (Sénégal).
Chan EH, Chua TE (1980) Reproduction in the greenback grey mullet, Liza subviridis (Valenciennes, 1836. J Fish Biol 16:505–519
Chavance P, Flores-Coto C, Sanchez-Iturbe A (1984) Early life history and adult biomass of sea bream in the Terminos Lagoon, southern Gulf of Mexico. Trans Am Fish Soc 113:166–177
Cubillos LA, Alarcón C, Castillo-Jordán C, Claramunt G, Oyarzún C, Gacitúa S (2011) Spatial and temporal changes in batch fecundity of common sardine and anchovy off central and southern Chile (2002–2007). Ciencias Mar 37:547–564
Dadzie S, Abou-Seedo F, Manyala JO (2008) Length–length relationship, length–weight relationship, gonadosomatic index, condition factor, size at maturity and fecundity of Parastromateus niger (Carangidae) in Kuwaiti waters . J Appl Ichthyol 24:334–336
Dagang C, Changan L, Shuozeng D (1992) The biology of flatfish (Pleuronectinae) in the coastal waters of China. Netherlands J sea Res 29:25–33
Dalzell P (1985) Some aspects of the reproductive biology of Spratelloides gracilis (Schlegel) in the Ysabel Passage, Papua New Guinea. J Fish Biol 27:229–237
Davis TLO (1984) Estimation of fecundity in barramundi, Lates calcarifer (Bloch), using an automatic particle counter. Mar Freshw Res 35:111–118
Delacy AC (1964) Maturation, gestation and birth of rockfish (Sebastodes) from Washington and adjacent waters.
DeMartini E, Anderson M (1980) Comparative survivorship and life history of painted greenling (Oxylebius pictus) in Puget Sound, Washington and Monterey Bay, California. Environ Biol Fishes 5:33–47
DeMartini EE, Fountain RK (1981) Ovarian cycling frequency and batch fecundity in the queenfish, Seriphus politus: attributes representative of serial spawning fishes. Fish Bull 79:547–560
Demirel N, Yüksek A (2013) Reproductive biology of Trachurus mediterraneus (Carangidae): a detailed study for the Marmara–Black Sea stock. J Mar Biol Assoc United Kingdom 93:357–364
Déniel C (1983) La reproduction des poissons plats (Téléostéens, Pleuronectiformes) en Baie de Douarnenez. 1. Cycles sexuels et fécondité des arnoglosses Arnoglossus thori, A. laterna, A. imperialis (Bothidae)= Reproduction of flatfishes in Douarnenez Bay: Sexual cycles and fecundity of Arnoglossus thori, A. laterna, A. imperialis. Cah Biol Mar
Drevetnyak K V, Gusev E V (1996) On fecundity of redfish (Sebastes mentella, Travin) from

the Norwegian-Barents Sea population. ICES C
Drevetnyak KV, Kluev AI (2005) On fecundity of Sebastes viviparus from the Northeast Arctic. ICES C
Eldridge MB, Jarvis BM (1995) Temporal and Spatial Variation in Fecundity of Yellowtail Rockfish . Trans Am Fish Soc 124:16–25
Elliott JM (1995) Fecundity and egg density in the redd for sea trout . J Fish Biol 47:893–901
Emerson LS, Walker MG, Witthames PR (1990) A stereological method for estimating fish fecundity . J Fish Biol 36:721–730
Evans R, Russ G, Kritzer J (2008) Batch fecundity of Lutjanus carponotatus (Lutjanidae) and implications of no-take marine reserves on the Great Barrier Reef, Australia. J Int Soc Reef Stud 27:179–189
de Eyto E, White J, Boylan P, Clarke B, Cotter D, Doherty D, Gargan P, Kennedy R, McGinnity P, O'Maoiléidigh N, O'Higgins K (2015) The fecundity of wild Irish Atlantic salmon Salmo salar L. and its application for stock assessment purposes . Fish Res 164:159–169
Facade SO, Olaniyan CIO (1972) The biology of the West African shad Ethmalosa fimbriata (Bowdich) in the Lagos Lagoon, Nigeria . J Fish Biol 4:519–533
Fazli H, Janbaz AA, Taleshian H, Bagherzadeh F (2008) Maturity and fecundity of golden grey mullet (Liza aurata Risso, 1810) in Iranian waters of the Caspian Sea. J Appl Ichthyol 24:610–613
Finucane JH, Collins LA, Brusher HA, Saloman CH (1986) Reproductive biology of king mackerel, Scomberomorus cavalla, from the southeastern United States. Fish Bull 84:841–850
Fleming IA (1998) Pattern and variability in the breeding system of Atlantic salmon (Salmo salar), with comparisons to other salmonids. Can J Fish Aquat Sci 55:59–76
Fontana A, Le Guen J-C (1969) Étude de la maturité sexuelle et de la fécondité de Pseudotolithus (fonticulus) elongatus.
Freitas MO, Freitas MO, Previero M, Previero M, Minte-Vera C V, Minte-Vera C V, Spach HL, Spach HL, Francini-Filho RB, Francini-Filho RB, Moura RL, Moura RL (2018) Reproductive biology and management of two commercially important groupers in the SW Atlantic . Environ Biol Fishes 101:79–94
Ganias K, Somarakis S, Machias A, Theodorou A (2004) Pattern of oocyte development and batch fecundity in the Mediterranean sardine . Fish Res 67:13–23
Gerasimchuk V V (1987) On the fecundity of Antarctic sidestripe, Pleuragramma antarcticum. J Ichthyol 28:98–100
Gesteira TCV (1972) Sobre a reprodução e fecundidade da serra, Scomberomorus maculatus (Mitchill), no Estado do Ceará.
Gesteira TCV, Rocha CAS (1976) Estudo sobre a fecundidade do ariacó, Lutjanus synagris (Linnaeus), da costa do Estado do Ceará (Brasil).
Gladstone W, Westoby M (1988) Growth and reproduction in Canthigaster valentini (Pisces, Tetraodontidae): a comparison of a toxic reef fish with other reef fishes. Environ Biol Fishes 21:207–221
Grande M, Murua H, Zudaire I, Korta M (2010) Spawning activity and batch fecundity of skipjack, Katsuwonus pelamis, in the Western Indian Ocean. IOTC-2010 AZTI Tecnalia, Portualde z/g
Grimes CB, Huntsman GR (1980) Reproductive biology of the vermilion snapper, Rhomboplites aurorubens, from North Carolina and South Carolina. Fish Bull 78:137
Gundersen AC, Kjesbu OS, Nedreaas KH, Stene A (1999) Fecundity of northeast Arctic Greenland halibut (Reinhardtius hippoglossoides). J Northwest Atl Fish Sci 25:29–36

Gundersen AC, Nedreaas KH, Kjesbu OS, Albert OT (2000) Fecundity and recruitment variability of Northeast Arctic Greenland halibut during 1980–1998, with emphasis on 1996–1998. J Sea Res 44:45–54

Gundersen AC, Rønneberg JE, Boje J (2001) Fecundity of Greenland halibut (Reinhardtius hippoglossoides walbaum) in East Greenland waters. Fish Res 51:229–236

Harrod C, Griffiths D (2004) Reproduction and fecundity of the Irish pollan (Coregonus autumnalis Pallas, 1776), a threatened lake coregonid. 117–124

He W-P, Li Y-X, Liu M, Radhakrishnan K V, Li Z-J, Murphy BR, Xie S-G (2011) Reproductive biology of Coilia mystus (Linnaeus) from the Yangtze Estuary, China: responses to overexploitation: Reproductive biology of Coilia mystus (Linnaeus) . J Appl Ichthyol 27:1197–1202

Healey MC (1971) Gonad development and fecundity of the sand goby, Gobius minutus Pallas. Trans Am Fish Soc 100:520–526

Hedeholm R, Grønkjær P, Rysgaard S (2011) Energy content and fecundity of capelin (Mallotus villosus) along a 1,500-km latitudinal gradient . Mar Biol 158:1319–1330

Heinimaa S, Heinimaa P (2004) Effect of the female size on egg quality and fecundity of the wild Atlantic salmon in the sub-arctic River Teno. Boreal Environ Res 9:55–62

Hickling CF (1970) A contribution to the natural history of the english grey mullets [Pisces, Mugilidae]. J Mar Biol Assoc United Kingdom 50:609–633

Hinckley S (1987) The reproductive biology of walleye pollock, Theragra chalcogramma, in the Bering Sea, with reference to spawning stock structure. Fish Bull 85:481–498

Hislop JRG, Hall WB (1974) The fecundity of whiting, Merlangius merlangus (L.) in the North Sea, the Minch and at Iceland. ICES J Mar Sci 36:42–49

Hourigan TF, Radtke RL (1989) Reproduction of the Antarctic fish Nototheniops nudifrons. Mar Biol 100:277–283

Ivo CTC (1974) Sobre a fecundidade da cavala, Scomberomorus cavalla (Cuvier), em águas costeiras do Estado do Ceará (Brasil).

Jons GD, Miranda LE (1997) Ovarian weight as an index of fecundity, maturity, and spawning periodicity. J Fish Biol 50:150–156

Jonsson B, Hindar K (1982) Reproductive strategy of dwarf and normal Arctic charr (Salvelinus alpinus) from Vangsvatnet Lake, western Norway. Can J Fish Aquat Sci 39:1404–1413

Joseph J (1963) Fecundity of yellowfin tuna (Thunnus albacares) and skipjack tuna (Katsuwonus pelamis) from the eastern Pacific Ocean. Inter-Amer Trop Tuna Comm Bull 7:255–292

Kacem H, Neifar L (2014) The reproductive biology of the grey triggerfish Balistes capriscus (Pisces: Balistidae) in the Gulf of Gabès (south-eastern Mediterranean Sea). J Mar Biol Assoc United Kingdom 94:1531–1537

Kavanagh KD (2000) Larval brooding in the marine damselfish Acanthochromis polyacanthus (Pomacentridae) is correlated with highly divergent morphology, ontogeny and life-history traits. Bull Mar Sci 66:321–337

Kelly KH, Stevenson DK (1985) Fecundity of Atlantic herring (Clupea harengus) from three spawning areas in the western Gulf of Maine, 1969 and 1982. J Northw Atl Fish Sci 6:149–155

Kjesbu OS (1988) Fecundity and maturity of cod (Gadus morhua L.) from northern Norway.

Kjesbu OS, Klungsøyr J, Kryvi H, Witthames PR, Walker MG (1991) Fecundity, atresia, and egg size of captive Atlantic cod (Gadus morhua) in relation to proximate body composition. Can J Fish Aquat Sci 48:2333–2343

Kock K-H, Kellermann A (1991) Reproduction in Antarctic notothenioid fish. Antarct Sci 3:125–150

Kock KH (1989) Reproduction in fish around Elephant Island. Arch FischWiss 39:171–210
Kokita T (2003) Potential latitudinal variation in egg size and number of a geographically widespread reef fish, revealed by common-environment experiments. Mar Biol 143:593–601
Koslow JA, Bell J, Virtue P, Smith DC (1995) Fecundity and its variability in orange roughy: effects of population density, condition, egg size, and senescence. J Fish Biol 47:1063–1080
Lajud NA, Astarloa JMD de, González-Castro M (2016) Reproduction of Brevoortia aurea (Spix & Agassiz, 1829)(Actinopterygii: Clupeidae) in the Mar Chiquita Coastal Lagoon, Buenos Aires, Argentina. Neotrop Ichthyol 14:
Lang ET, Fitzhugh GR (2015) Oogenesis and fecundity type of gray triggerfish in the Gulf of Mexico. Mar Coast Fish 7:338–348
Laroche JL, Richardson SL (1980) Reproduction of northern anchovy, Engraulis mordax, off Oregon and Washington. Fish Bull 78:603–618
Lear WH (1970) Fecundity of Greenland halibut (Reinhardtius hippoglossoides) in the Newfoundland–Labrador area. J Fish Board Canada 27:1880–1882
Lee CS, Hur YH, Lee JY, Kim WK, Hong SH, Hwang SJ, Choi SH (2005) Maturity and spawning of Pacific cod (Gadus macrocephalus) in the East Sea. Korean J Fish Aquat Sci 38:245–250
Li Y, Xie S, Li Z, Gong W, He W (2007) Gonad development of an anadromous fish Coilia ectenes (Engraulidae) in lower reach of Yangtze River, China. Fish Sci 73:1224–1230
Liao Y-Y, Luo S-R, Liu K-M (2014) Reproductive Biology of the Bombay-Duck Harpadon Microchir in the Coastal Waters off Southwestern Taiwan. J Mar Sci Technol 22:658–665
Lim H, Le M, An C, Kim S, Park M, Chang Y (2010) Reproductive cycle of yellow croaker Larimichthys polyactis in southern waters off Korea. Fish Sci 76:971–980
Lisovenko LA (1965) Sovetskie rybokhozyaistvennye issledovaniya v severo-vostochnoi chasti Tikhogo okeana. In: Moiseev P. (eds) Israel Program for Scientific Translations, Jerusalem, pp 162–169
Lisovenko LA, Sil'yanova ZS (1979) The fecundity of some species of the family Nototheniidae in the Atlantic sector of the Southern Ocean. J Ichthyol 19:79–85
Loewen TN, Gillis D, Tallman RF (2010) Maturation, growth and fecundity of Arctic charr, Salvelinus alpinus (L.), life-history variants co-existing in lake systems of Southern Baffin Island, Nunavut, Canada. Hydrobiologia 650:193–202
Love MA, McGowen GE, Westphal W, Lavenberg RJ, Martin L (1984) Aspects of the life history and fishery of the white croaker, Genyonemus lineatus (Sciaenidae), off California. Fish Bull 82:179–198
Love MS (1990) Life history aspects of 19 rockfish species (Scorpaenidae: Sebastes) from the Southern California Bight.
Love MS, Westphal W V (1981) Growth, reproduction, and food habits of olive rockfish Sebastes serranoides, off central California . Fish Bull 79:533–545
Ma Y, Kjesbu OS, Jørgensen T (1998) Effects of ration on the maturation and fecundity in captive Atlantic herring (Clupea harengus). Can J Fish Aquat Sci 55:900–908
Macchi GJ, Acha ME, Lasta CA (1996) Desove y fecundidad de la corvina rubia Micropogonias furnieri Desmarest, 1823 del estuario del Río de la Plata, Argentina. Boletín Inst Español Oceanogr 12:99–113
Macewicz BJ, Hunter JR (1993) Spawning frequency and batch fecundity of jack mackerel, Trachurus symmetricus, off California during 1991. CalCOFI Rep 34:112–121
Marteinsdottir G, Begg GA (2002) Essential relationships incorporating the influence of age, size and condition on variables required for estimation of reproductive potential in

Atlantic cod Gadus morhua. Mar Ecol Prog Ser 235:235–256
Martins AS, Haimovici M (2000) Reprodution of the cutlassfish Trichiurus lepturus in the southern Brazil subtropical convergence ecosystem.
May AW (1967) Fecundity of Atlantic Cod. J Fish Res Board Canada 24:1531–1551
Mayer I, Shackley SE, Witthames PR (1990) Aspects of the reproductive biology of the bass, Dicentrarchus labrax L. II. Fecundity and pattern of oocyte development. J Fish Biol 36:141–148
McBride RS, Thurman PE (2003) Reproductive biology of Hemiramphus brasiliensis and H. balao (Hemiramphidae): maturation, spawning frequency, and fecundity. Biol Bull 204:57–67
Mena-Loría AR (2009) No. Instituto Politecnico Nacional
La Mesa M, Caputo V, Eastman JT (2008) The reproductive biology of two epibenthic species of Antarctic nototheniid fish of the genus Trematomus. Antarct Sci 20:355–364
Miñano M (1968) Estudio de la fecundidad y ciclo sexual de la anchoveta (Egraulis ringens. J.) en la zona de Chimbote.
Minto C, Nolan CP (2006) Fecundity and maturity of orange roughy (Hoplostethus atlanticus Collett 1889) on the Porcupine Bank, Northeast Atlantic. Environ Biol Fishes 77:39–50
Moresco A (2006) Biologia reprodutiva do peixe-rei Odontesthes argentinensis (Atherinopsidae), da região da marinha costeira e região estuarina da Lagoa dos Patos.
Morse WW (1981) Reproduction of the summer flounder, Paralichthys dentatus (L. J Fish Biol 19:189–203
Nagelkerken WP (1979) Biology of the graysby, Epinephelus cruentatus, of the coral reef of Curaçao. Stud fauna Curaçao other Caribb Islands 60:1–118
Oosthuizen E, Daan N (1974) Egg fecundity and maturity of North Sea cod, Gadus morhua. Netherlands J Sea Res 8:378–397
Pájaro M, Macchi GJ, Sánchez RP (1997) Fecundidad y frecuencia reproductiva de las poblaciones bonaerense y patagónica de anchoíta Argentina Engraulis anchoita.
Perea de la Matta Á, Buitrón Díaz B (2004) Fecundidad, frecuencia de desove y estado de madurez gonadal de la anchoveta peruana durante el invierno austral 2001.
Peterson CL (1961) Fecundity of the anchoveta (Cetengraulis mysticetus) in the Gulf of Panama.
Phillips JB (1964) Life history studies on ten species of rockfish (genus Sebastodes). Calif Dep Fish Game Fish Bull 126:
Pinheiro PB (2004) Biologia reproductiva do peixe-re, Elagatis bipinnulata (Quoy & Gaimard, 1825), capturado na Zona Economica Exclusiva (ZEE) do nordeste do Brasil. Universidade Federal de Pernambuco
Power M, Dempson JB, Reist JD, Schwarz CJ, Power G (2005) Latitudinal variation in fecundity among Arctic charr populations in eastern North America. J Fish Biol 67:255–273
Rae GA, Calvo J (1995) Fecundity and reproductive habits in Patagonotothen tessellata (Richardson, 1845) from the Beagle Channel, Argentina. Antarct Sci 7:235–240
Rijnsdorp AD (1991) Changes in fecundity of female North Sea plaice (Pleuronectes platessa L.) between three periods since 1900. ICES J Mar Sci 48:253–280
Rodríguez JA, Villegas JP, Chavarría AL (1986) Fecundidad y época de desove del arenque hebra opisthonema libertate (Pisces Clupeidae) en el Golfo de Nicoya, Costa Rica. Uniciencia 3:87–93
Ross JL, Merriner J V (1983) Reproductive biology of the blueline tilefish, Caulolatilus microps, off North Carolina and South Carolina. Fish Bull 81:553
Ross SW (1984) Reproduction of the banded drum, Larimus fasciatus. North Carolina Fish Bull 82:227–235

Roumillat WA, Brouwer MC (2004) Reproductive dynamics of female spotted seatrout (Cynoscion nebulosus) in South Carolina.

Saborido-Rey F, Domínguez-Petit R, Garabana D, Sigurðsson Þ (2015) Fecundity of Sebastes mentella and Sebastes norvegicus in the Irminger Sea and Icelandic waters. Ciencias Mar 41:107–124

Schmidt K (2014) N. California State University, Monterrey Bay

Shepherd GR, Grimes CB (1984) Reproduction of weakfish, Cynoscion regalis, in the New York Bight and evidence for geographically specific life history characteristics. Fish Bull 82:501

de Souza JN, Giamas MTD, Junior HV (1988) Tipo de desova e fecundidade em Anchoviella lepidentostole (Fowler, 1911). Rev da Fac Med Veterinária e Zootec da Univ São Paulo 25:251–260

Souza L de LG, Chellappa S, Gurgel H de CB (2007) Biologia reprodutiva do peixe-donzela, Stegastes fuscus Cuvier, em arrecifes rochosos no nordeste do Brasil. Rev Bras Zool 24:419–425

St-Pierre J-F, De Lafontaine Y (1995) Fecundity and reproduction characteristics of beaked redfish (Sebastes fasciatus and S. mentella) in the Gulf of St. Lawrence. Citeseer,

Thomson JA (1962) On the fecundity of Pacific cod (Gadus macrocephalus Tilesius) from hecate strait, British Columbia. J Fish Board Canada 19:497–500

Trippel EA, Neil SRE (2004) Maternal and seasonal differences in egg sizes and spawning activity of northwest Atlantic haddock (Melanogrammus aeglefinus) in relation to body size and condition. Can J Fish Aquat Sci 61:2097–2110

Turnpenny AWH, Bamber RN, Henderson PA (1981) Biology of the sand-smelt (Atherina presbyter Valenciennes) around Fawley power station. J Fish Biol 18:417–427

Vazzoler AEA de M (1963) Sobre a fecundidade e a desova da pescada-foguete. Bol do Inst Ocean 13:33–40

Veerappanm N, Ramanathan M, Ramaiyan V (1997) Maturation and spawning biology of Amblygaster sirm from Parangipettai, southeast coast of India. J Mar Biol Assoc India 39:89–96

Veith WJ (1979) Reproduction in the live-bearing teleost Clinus superciliosus. African Zool 14:208–211

Vieira ARS (2007) N. Universidade de Lisboa

Washington PM, Gowan RE, Ito DH (1978) A biological report on eight species of rockfish (Sebastes spp.) from Puget Sound, Washington. US Department of Commerce, Northwest and Alaska Fisheries Center,

Zaki S, Jayabalan N, Al-Kiyumi F, Al-Kharusi L, Al-Habsi S, Al-Marzouqi A (2011) Fishery, biology and population dynamics of three small pelagic fish species (Indian oil sardine Sardinella longiceps, Indian mackerel Rastrelliger kanagurta and Indian scad Decapterus russelli) from the Sultanate of Oman. Proj Final Report-Part II

Season length references

Almada VC, Carreiro HAV, Faria CBM de, Gonçalves EJ (1996) The breeding season of Coryphoblennius galerita in Portuguese waters. J Fish Biol 295–297

Bowers AB (1954) Breeding and growth of whiting (Gadus merlangus L.) in Isle of Man waters. J Mar Biol Assoc United Kingdom 33:97–122

Buchanan-Wollaston H (1914) Report on the spawning-grounds of the plaice in the North Sea, 1911. Fish Invest Board Agric Fish 2:18

Bushnell M (2007) Reproduction of Zebrasoma flavescens: oocyte maturation, spawning patterns, and an estimate of reproductive potential for female yellow tang in Hawai‘i.

Chiarella LA, Conover DO (1990) Spawning season and first-year growth of adult bluefish from the New York Bight. Trans Am Fish Soc 119:455–462

Cook GS (2011) Changes in otolith microchemistry over a protracted spawning season influence assignment of natal origin. Mar Ecol Prog Ser 423:197–209

Coombs SH, Halliday NC, Southward AJ, Hawkins SJ (2005) Distribution and abundance of sardine (Sardina pilchardus) eggs in the English Channel from Continuous Plankton Recorder sampling, 1958-1980. Mar Biol Assoc United Kingdom J Mar Biol Assoc United Kingdom 85:1243

Corbin Pg (1947) The spawning of mackerel, Scomber scombrus L., and pilchard, Clupea pilchardus Walbaum, in the Celtic Sea in 1937–39. J Mar Biol Assoc United Kingdom 27:65–132

Craig PC (1998) Temporal spawning patterns of several surgeonfishes and wrasses in American Samoa.

Damas D (1909) Contribution a la biologie des Gadides. Cons Perm Internat Explor Mer, Rapp Proc-Verb 10:1–277

Domínguez-Petit R, Saborido-Rey F, Medina I (2010) Changes of proximate composition, energy storage and condition of European hake (Merluccius merluccius, L. 1758) through the spawning season. Fish Res 104:73–82

Ehrenbaum E (1909) Eier und Larven von Fischen. Nord Plankt 217–413

Gause GF (1934) The struggle for existence .

Graham M (1924) The annual cycle in the life of the mature cod in the North Sea. Min Agric Fish, Fish Inverst 6:1–77

Hickling C (1930) The natural history of the hake. Part III. Min Agric Fish, Fish Inverst 12:1–78

Hickling C (1945) The seasonal cycle in the Cornish pilchard, Sardina pilchardus Walbaum. Ibid 26:115–138

Hodgson W (1929) Investigations into the age, length, and maturity of the herring of the southern North Sea. Part III. Min Agric Fish, Fish Inverst 11:1–75

Jenkins T (1950) Fishes of the British Isles. Warne & Co., London

Kokita T (2003) Potential latitudinal variation in egg size and number of a geographically widespread reef fish, revealed by common-environment experiments. Mar Biol 143:593–601

Kritzer JP (2004) Sex-specific growth and mortality, spawning season, and female maturation of the stripey bass (Lutjanus carponotatus) on the Great Barrier Reef. Fish Bull 102:94–107

LaPlante LH, Schultz ET (2007) Annual fecundity of tautog in Long Island Sound: size effects and long-term changes in a harvested population. Trans Am Fish Soc 136:1520–1533

Loher T, Seitz A (2008) Characterization of active spawning season and depth for eastern Pacific halibut (Hippoglossus stenolepis), and evidence of probable skipped spawning. J

Northwest Atl Fish Sci 41:
Al Mahmud N, Rahman HM, Mostakim GM, Khan M, Quader G, Shahjahan M, Lucky NS, Islam MS (2016) Cyclic variations of gonad development of an air-breathing fish, Channa striata in the lentic and lotic environments. Fish Aquat Sci 19:1–7
Matsuura Y (1996) A probable cause of recruitment failure of the Brazilian sardine Sardinella aurita population during the 1974/75 spawning season. South African J Mar Sci 17:29–35
McDonough CJ, Roumillat WA, Wenner CA (2003) Fecundity and spawning season of striped mullet (Mugil cephalus L.) in South Carolina estuaries.
McMichael RH, Peters KM (1989) Early life history of spotted seatrout, Cynoscion nebulosus (Pisces: Sciaenidae), in Tampa Bay, Florida. Estuaries 12:98–110
Milton P (1983) Biology of littoral blenniid fishes on the coast of south-west England. J Mar Biol Assoc United Kingdom 63:223–237
Moku M, Tsuda A, Kawaguchi K (2003) Spawning season and migration of the myctophid fish Diaphus theta in the western North Pacific. Ichthyol Res 50:52–58
Monteiro N, Almada VC, Santos AM, Vieira MN (2001) The breeding ecology of the pipefish Nerophis lumbriciformis and its relation to latitude and water temperature. J Mar Biol Assoc United Kingdom 81:1031–1033
Pampoulie C (2001) Demographic structure and life history traits of the common goby Pomatoschistus microps (Teleostei, Gobiidae) in a Mediterranean coastal lagoon (Rhône River delta, France). Acta Oecologica 22:253–257
Powell AB (2003) Larval abundance, distribution, and spawning habits of spotted seatrout (Cynoscion nebulosus) in Florida Bay, Everglades National Park, Florida.
Qasim SZ (1956) Time and Duration of the Spawning Season in some Marine Teleosts in Relation to their Distribution. Extr Du J Du Cons Int Pour L'exploration La Mer 21:141–155
Robertson DR (1990) Differences in the seasonalities of spawning and recruitment of some small neotropical reef fishes. J Exp Mar Bio Ecol 144:49–62
Sogabe A, Mohri K, Shoji J (2012) Reproductive seasonality of the seaweed pipefish Syngnathus schlegeli (Syngnathidae) in the Seto Inland Sea, Japan. Ichthyol Res 59:223–229
Tsikliras AC, Antonopoulou E (2006) Reproductive biology of round sardinella (Sardinella aurita) in north-eastern Mediterranean. Sci Mar 70:281–290
Tucker Jr JW, Campbell SW (1988) Spawning season of common snook along the east central Florida coast. Florida Sci 1–6
Watanabe S, Watanabe Y (2001) Brooding season, sex ratio, and brood pouch development in the seaweed pipefish, Syngnathus schlegeli, in Otsuchi Bay, Japan. Ichthyol Res 48:155–160
Wheeler AC (1969) The fishes of the British Isles and north-west Europe. Macmillan,
Williams AJ, Davies CR, Mapstone BD (2006) Regional patterns in reproductive biology of Lethrinus miniatus on the Great Barrier Reef. Mar Freshw Res 57:403–414
