# Supplementary material for: Life history optimisation drives latitudinal gradients and responses to global change in marine fishes
Source: PLoS Biol. 2023 May 25;21(5):e3002114. doi: 10.1371/journal.pbio.3002114 (PMC10212075; doi:10.1371/journal.pbio.3002114)
Supplement: Attachment — Submitted filename: Response to reviewers.pdf [file pbio.3002114.s024.pdf]

Response to reviewers

We are very thankful for the constructive feedback provided by the three reviewers and the Academic Editor. Their main concerns were: 1) the assumption that production scales at 2/3, 2) that the models estimating the demographic predictors did not account for phylogenetic relatedness, 3) that the differences between intra- and interspecific patterns were not clear, 4) that the details of the reproductive metrics and how the demographic parameter were estimated were unclear. We summarise how we have addressed these concerns below, and then address each of the reviewers' comments.

1) Regarding the scaling of production, we modified the life-history model in Day and Taylor (1997) to assume a scaling of ¾ and show that the results are qualitatively unchanged by this assumption. (Lines 302-305, Eqs. 4 & S2)

2) For the phylogenetic relatedness, we included a random effect of phylogeny with a covariance matrix reflecting the phylogenetic tree extracted using the R package 'rotl'. To optimise age at maturity in the life-history model, we used the posterior draws of the phylogenetically controlled models of growth, offspring size, and mortality. Optimal age at maturity and the related reproductive scaling were still predicted to increase with latitude see Figure 1 below). The phylogenetic signal (equivalent to Pagel's λ) was 0.19 for growth, 0.27 for offspring size, and 0.78 for mortality. (Lines 393-404, Table S1). We include a phylogeny effect for growth and offspring size in the new analyses.

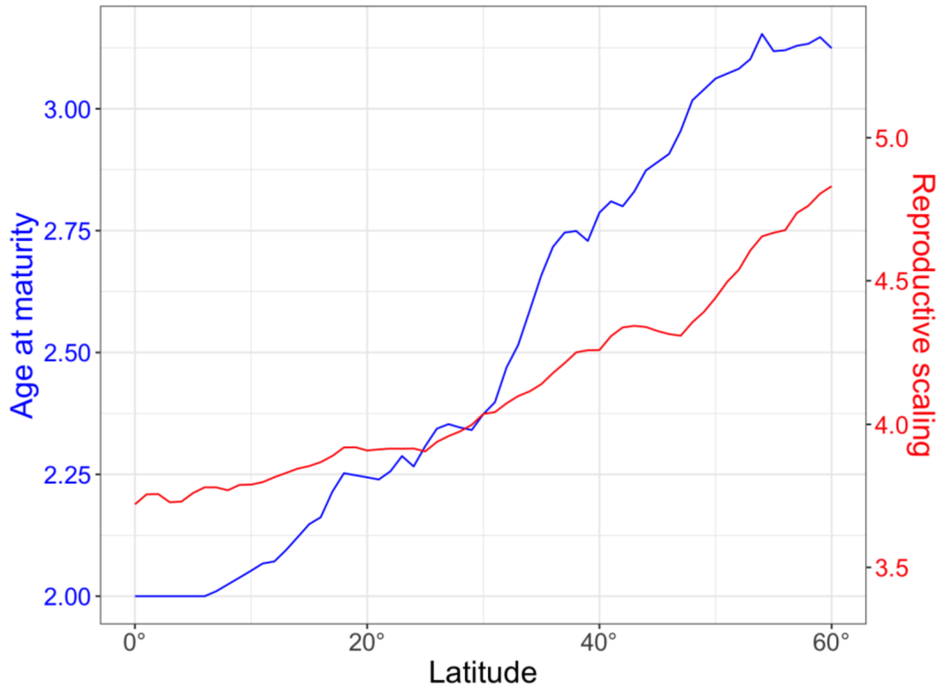

Optimal age at maturity (in blue, left-hand axis) and its corresponding reproductive scaling (in red, right-hand axis) using phylogenetically controlled estimates of growth, mortality, and offspring size.

While our results were robust to including a phylogeny effect for mortality, we think including such an effect is inappropriate for this variable specifically because our goal here was to understand the spatial patterns of mortality rates (regardless of evolutionary history) in order to apply those rates as ecological conditions (rather than evolutionary outcomes) in our model. We're not interested in the evolutionary association between latitude and mortality (which is what the phylogenetically-controlled analysis approximates), we are just seeking to understand what the likely mortality rate of the 'average fish is for a given latitude'. It is this latitude-specific mortality which we are interested in as an input to our model. Importantly, our findings are unaffected by whether we analyse mortality in a phylogenetically-controlled framework or not. Including a phylogeny effect here risks misestimating the ecological conditions of different latitudes due to potential covariances between latitude and phylogeny. Nevertheless, we defer to editorial preference here.

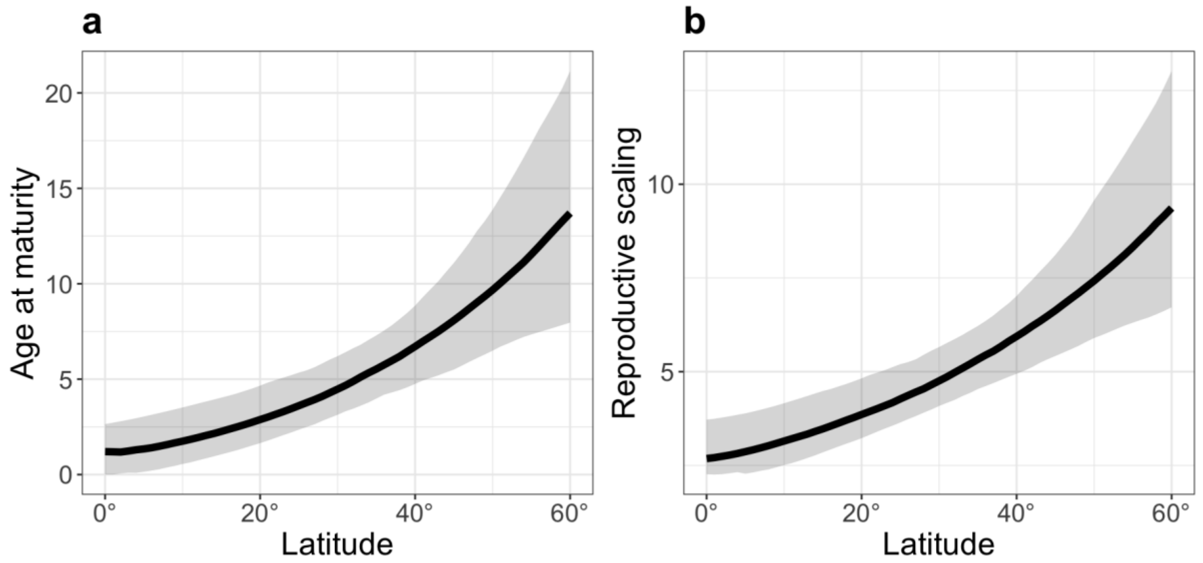

Optimal age at maturity (Panel a) and its corresponding reproductive scaling (Panel b) using phylogenetically controlled estimates of growth and offspring size but not mortality. The black lines show median estimates, and the ribbons show the 95% credible intervals.

3) In regards to the differences between intra- and interspecific patterns – this is an important point. Our study focuses mainly on among-species patterns in within-species relationships. That's because most of our data only include a single latitude for a given species – hence we can only calculate fecundity scaling for that species at that latitude. Nevertheless, our model makes predictions about reproductive scaling both among- and within-species. In this revised version we therefore examined within-species patterns in fecundity scaling for the six species for which there are data at multiple latitudes and find that reproductive scaling also increases with latitude within species –so our model has more predictive power than we had appreciated. More generally, we now make it very clear when we are referring to within-species and among-species patterns to avoid confusing the reader. (Lines 112-120, Fig. S3)

4) We have also clarified that we used batch fecundity as our metric for fecundity. Other measurements of fecundity, such as annual fecundity, are orders of magnitude less common

in the literature and therefore getting estimates of reproductive scaling across latitudes with such metrics is impossible. Moreover, populations for which there are data on both the number and size of eggs, as well as covariance with mass are very rare. So, batch fecundity is an incomplete measure but remains the best we have. Importantly, for our results to be nullified, there would need to be strong, negative latitudinal covariance in body size-egg size relationships, or spawning frequency and body size -there is no evidence for such negative covariance and some evidence for positive covariances. We now discuss these issues in detail in the manuscript. We also provide more exposition on how the demographic parameters were estimated. (Lines 378-380, 405-412)

Academic Editor

The Academic Editor felt that there was value in leaving the door somewhat open for a future submission, but thought that this would be "almost a new manuscript" - specifically, they stressed the need for:

1. making explicit that latitudinal variation in life history has been intensively studied

Agreed, we now make clear that latitudinal variation in some life history traits has been studied extensively (e.g. among and within species patterns in body size, offspring size) but other traits (e.g. among-species patterns in within-species fecundity scaling, or among species patterns in age at maturity, both which are essential inputs for the management of global fisheries), have received almost no attention. (Lines 59-63)

2. new analyses to separate intraspecific from interspecific analyses,

Agreed, we now make clear when our analyses are of interspecific patterns in intraspecific scaling relationships, when they are among-species model inputs (e.g. mortality), and when they are when they are among-species model outputs (e.g. age at maturity). (Lines 112 -116, Fig. S3)

3. accounting for phylogenetic relatedness among species in interspecific analyses as currently done in comparative analyses across species,

As noted above, we favour using non-phylogenetically controlled analyses of mortality (though our results are unchanged regardless of the analyses used), but all other analyses for all the other traits are strictly phylogenetically controlled. (Lines 393-404, Table S1).

4. more information about the exact metrics of reproduction used and providing justification for using them,

Agreed, we provided much more justification – basically these are the only data that are available for more than handful of species. We also make clear our conclusions are likely to be robust to more information about spawning frequency. (Lines 125-238, 378-380, 405-412)

5. more information about how life history traits were estimated (i.e. data used and account for confounding factors)

Agreed, we now provide much more information about these estimations. (Lines 336-415)

6. toning down the take-home message that reproduction is hyperallometric,

We never intended the take home message to be that reproduction is hyperallometric, rather we sought to explore why and how reproductive scaling varies (though an outcome of our model and analysis is that hyperallometry is predicted to be the rule in this group at least). We've modified the Discussion accordingly.

7. proposing mechanisms leading to hyperallometry

We now provide much more exposition on the mechanisms that lead to hyperallometry and what drives the patterns in hyperallometry. The simplest way of putting this is: whenever the scope for biological production increases with body size, and when an organism shifts

allocation from growth to reproduction, hyperallometry is inevitable. Remarkably, this mathematical inevitability was intuited by D'Arcy Thompson ~100 years ago but never formalised until now. The reason most other theories of growth don't predict hyperallometry is they assume the scope for production decreases with size (MTE, most DEB, GOLT), and that the relative allocation to reproduction and growth never changes with size. These assumptions have much less empirical support. (Lines 160-182, Fig. 3)

The biological mechanism that yields hyperallometry is therefore very simple. Whenever organisms allocate an increasing fraction of production to reproduction, increases in reproduction come at the expense of growth – hence reproduction increases more rapidly than does size, the very definition of hyperallometry. We now provide a detailed schematic showing this mechanism.

The mechanism that yields spatial patterns in hyperallometry that we observe is slightly more complex. In essence, any delay in the onset of reproduction will yield larger sizes at maturity and a shallower rate of increase in the size-production relationship (assuming size scales with scope for production <1). Hence, when an organism that has delayed reproduction does finally start allocating to reproduction, growth rate declines rapidly because the size-production relationship is relatively shallow at these later ages and larger sizes (because production scales ***hypoallometrically****), hence the rate of increase in reproduction is much greater in a relative sense than rate of growth – thus hyperallometry should be steeper in organisms that delay reproduction for longer and this is exactly what we observe.*
We now provide much more exposition on these mechanisms.

8. justifying the 2/3 scaling power

This is a great point, we now show that our results do not depend on the use of 2/3, we get the same result qualitatively regardless of the scaling relationship.

9. making the connection with the slow-fast continuum of life history variation]

This is another great point, we now make this connection clear. (Lines 151-153).

Reviewer #1:

My major concern is about the contribution of this study to our understanding of the hyperallometric reproduction scaling. This is a theoretical modeling study. In my opinion, a theoretical model may make contributions in two related ways. By employing empirical parameters, the model makes quantitative predictions, which agree with the measured values; or it explains qualitatively and conceptually what the underlying mechanisms of a phenomenon are.

After reading the manuscript, I asked myself what I learned. Is it the hyperallometric scaling? We already knew it from previous studies. We don't need this study to tell us this. The model's quantitative predictions on the scaling powers are not accurate, and the manuscript did not make the mechanisms clear either.

The authors pointed out that the model predicts "tropical fish start life at (1) a small size, (2) grow rapidly, and (3) suffer high mortality. Consequently, tropical fish maximise their fitness by diverting energy into (4) reproduction earlier in life, reaching smaller sizes, (5) having only shallow reproductive scaling exponents."

From many previous theoretical studies, we already have pretty good understandings on how temperature affects size, growth rate, age at maturation…(point 1-4). None of those is new.

We appreciate the reviewer's point here - other explanations for why body size and temperature might covary have been proposed but we would argue that our explanation, focused on a formal life history optimisation approach is relatively new. The idea that life history optimisation could drive some of the fundamental patterns we observe in nature is increasingly well appreciated but under explored (for example, see a recent paper by some of the authorship team in Science *this year showing that optimisation may explain metabolic scaling ectotherms). Essentially, we're arguing for a new, competing explanation for well-known temperature relationships – one that doesn't invoke other, more physiologically-focused explanations. But we also agree that there are components to our study that are completely novel – that life history optimisation shapes latitudinal patterns in age at maturity and previously unrecognised patterns in reproductive scaling*

But, the target of this study, the fifth, the mechanism of the reproductive hyperallometry is not explained in the manuscript. At least, after reading the manuscript, I still don't know what the mechanism underlying the hyperallometry is, and I am still fuzzy about how higher temperature leads to a shallow scaling slope. From the current version, I don't have a qualitative and intuitive understanding of it. I only know the model predicts it, but why and how? What are the "biological" and/or "evolutionary" reasons hidden in the model that lead to this hyperallometry?

This is a really great point – we now see that we didn't walk the reader through how hyperallometry arises (see our response to the editor at point 7) and we now provide exposition in the main text as well. (Lines 378-380, 405-412)

Similarly, another important prediction, the covariance between reproductive scaling and age at maturity, lacks a mechanistic explanation too.

In the discussion, the authors simply described the results, but didn't give any explanation why.

Again, in my opinion, if a model fails to make accurate quantitative predictions, as this model does, at least it should offer some qualitative insight into the question.

This is a fair point – we've now gone into much more detail as to how and why scaling and age at maturity covary. (Lines 164-178)

I also have a few minor suggestions.

1. In the introduction and abstract, the authors kept using the word "reproduction." I am worried that some readers may get confused: is it reproduction rate (per unit time)? or lifetime biomass reproduced? Or lifetime number of offspring….? Please use one sentence to clarify at the beginning.

We were referring to batch fecundity. We have clarified this now. (Lines 123-28, 369-370)

2. The 2/3 scaling power of the production function comes from nowhere. I have no problem with the 2/3 power, but can the authors give some evidence supporting this power. If no data is available, can the authors give some qualitative explanations? And, in the

supplementary, I think the authors need to perform some sensitivity test to check how robust the results are, if they change the scaling power.

We do not have evidence supporting this power, indeed there is no good understanding of what the scaling of production is though it seems likely that it is around the same scaling as metabolism (it seems unlikely that the rate of production could proceed faster than the metabolic rate). As suggested by another reviewer, we have rerun the optimisation assuming a ¾ power scaling, with qualitatively the same predictions. (Eqs. 1 & 4, Lines 302-305)

3. From equation (2), the fecundity has a unit in mass (gram, kg…). But, in the figures, it is measured in number of eggs. What is the assumption under this conversion? And this is related to my next question.

We address this in your next point.

4. The authors admitted that "the values of reproductive scaling estimated from the data are lower than those predicted by the life-history model." And they offered a possible reason: "However, because egg size strongly increases with latitude, reproduction measured as egg volume instead of egg number would scale steeper with mass, and the scaling would increase more rapidly with increasing latitude."

This sounds like the study is not finished. Why didn't they use egg volume (or weight) then? No data? I believe some data are available. And since the equation (2) uses weight, not number, it seems more appropriate to use mass/volume.

The data at the appropriate scale are indeed lacking. In order to estimate these relationships appropriately, we need the covariation between body size, egg number and egg weight for the

same population. Unfortunately, very few such studies exist and are concentrated around just a few degrees of latitude. (Lines 125-238, 378-380, 405-412)

Reviewer #2:

[identifies himself as Charles A. S. Hall]

This is a relatively sophisticated, clearly written, well documented and certainly interesting manuscript, or at least it appears so to me, who might not be the best qualified reviewer. There is much about it that is professional and very good. It could be published more or less as is.

We are pleased to see the manuscript was well received by this reviewer.

Nevertheless there are some quite fundamental issues where I have some problems. Perhaps it comes from differences in our backgrounds and training, although I have some experience with similar (but not the same) questions and some experience with fish. First, I am not sure that the conclusions are so novel (see my comment for page 9).

We are unaware of studies investigating how or why reproductive scaling exponents vary in space, nor why they covary with age at maturity.

Second, if I were to try to determine the latitudinal restriction on the production of propagules (p 14) I would start with temperature which the authors bring up but do not explore on page 16. Why not? Maybe they think it not important for their questions. If so they should state that.

We wanted to quantify how demography changed across space and, while temperature is one of the main environmental factors changing across latitudes, it is not the only one and we cannot be certain that it drives the spatial variation in mortality, the main driver of differences in studied life-history traits. Therefore, we did not want to assign causality based on correlation.

The use of 2/3 for a mass parameter needs justification.

We agree that the scaling of 2/3 is arbitrary and was only chosen because Day and Taylor 1997 used this value. Given this suggestion and suggestions from the editor, we have reanalysed the life-history optimisation model now assuming a scaling of ¾. Our conclusions are unchanged. (Lines 302-305, Eqs. 4 & S2)

There are some additional almost philosophical questions that might undermine the logic of this paper but have not been well resolved in all biology. First, do we know the value of fecundity? Jim Brown and colleagues have been exploring this issue and deriving some surprising results, or maybe they are assumptions. First he starts with the observation that (in the absence of human large effects) in most organisms over a number of generations two adults generate two offspring, year after year, whether a shark or an equal sized tuna. I fought this but think now it not a bad assumption. SO what is the advantage of high fecundity? I can think of two possibilities: with large environmental change this allows expansion into new habitat and possibly it allows greater evolutionary fine tuning -implying that tunas are more "fit" than sharks (and Osteichthyes may be taking over from Chondrichthyes over long time.) This also gets into all kinds of unresolved , (or barely resolved if you like Don Strong) questions of density dependance. You might send

him a letter.

What this would mean for this paper is that there may be little or no advantage in large fecundity, be it with latitude or age or what have you. That certainly goes against all I have assumed for the past 50+ years but there you have it. Do the authors want to touch that? What would be the implications?   I do not require it, but it might strengthen their paper. Overall interesting, well done paper.

This is a really interesting point. The assumption that, in the long run, population growth rates sit at around replacement, is reasonable (indeed some of us are on papers making such assumptions). But this issue doesn't negate the idea that different environments impose different selection regimes on life-histories and that life histories must therefore evolve. For example, early mortality in fish is usually very large and, under different conditions egg size and egg number have different optima (1). *An important distinction here is about absolute versus relative fitness, as well as hard and soft selection – populations may not be growing but there will still be selection for the 'best' phenotype/life history within that population. Hence, while the assumption that λ=1 can be useful for thinking about trait evolution more generally, it feature less when thinking about trait evolution in response to different selection regimes (as we do here). Given other readers might also be curious about this issue, we now deal with it briefly in the revised ms and cite the Brown et al. paper accordingly. (Lines 201-202)*

Brown, James H., Charles A.S. Hall and Richard M. Sibly.   2018. Energy and Fitness: all species are equally fit. Nature Ecology and Evolution: Vol. 2: February: 262-268.

Specifics:

9-17 "The recent discovery of that reproduction is often hyperallometric, whereby reproductive output increases disproportionately with body mass, contradicts most theories´ not for someone who studied cod fish in his youth. Seems like if you make it to a certain size)and mortality for most critters is size dependent) than why not start putting net energy into eggs vs size? Seemed logical to me 60 years ago. Didn't Dick Wiegert say that? Lamont Cole (maybe not)

The idea that reproduction is hyperallometric is completely new to some, but axiomatic to others. Beverton and Holt suspected that larger mothers contributed disproportionately as did early empiricists but as Table S1 in Barneche et al. 2018 shows, almost all theory and most fisheries models explicitly assume that size and reproduction are strictly linear (some, e.g. Andersen et al. even assume that larger mothers have relatively lower reproductive outputs – hypoallometry). We recently demonstrated that this causes manifold problems for fisheries management (Marshall et al. 2021 PNAS). We now acknowledge that this idea has been around in some form for a long time but most formal theory and almost all management has assumed otherwise. (Lines 33- 39)

10-59 such a test

Corrected

14 136 fewer offspring (or less reproductive output)

colder fish are far more137

'efficient' at producing offspring (both in terms of size and number) for a given female mass -- well, at a first cut. Wouldn't you expect an organism to be more efficient at a colder temperature because respiration would be lower? What am I missing?

Lower temperature would also slow down metabolism and hence feeding and probably growth. I am surprised that more attention is not paid to temperature. (I see this mentioned but not considered on page 16 -why is it swept under the rug?

We've changed it to " colder fish produce far more offspring for a given female mass than smaller, warmer fish."

Page 15. Impact of fisheries/conservation areas seems probably correct and enhance importance of this paper.

We agree with the reviewer that our results have important implications for fisheries management.

19 250 I think that Jim Brown, who has thought a great deal about this, would argue for ¾. Not 2/3 as the size parameter. In fact its been a while since I have seen 2/3. Can you justify that?

We agree that the scaling of power is unknown and there is no strong evidence for a scaling value. We reran the analysis assuming a scaling of ¾ and the results were consistent. (Lines 302-305, Eqs. 4 & S2)

Lots of scatter in data that is probably telling us something,, but what?

This point got us thinking that we failed to emphasise that our model predicts a previously unrecognised biogeographical pattern despite having very few parameters – we now explore this a little more. (Lines 141-146)

Reviewer #3:

[identifies himself as Douglas S. Glazier]

General comments:

The authors show that the body-mass scaling exponent (slope) for fecundity (egg number per clutch) increases significantly with increasing latitude, a remarkable pattern. They also attempt to explain this result using a life-history optimization model, another noteworthy contribution. However, I have three major concerns with the present manuscript.

1) The authors do not clearly distinguish intra- vs. interspecific scaling patterns. In fact, their analysis appears to combine (confound) the two. This is problematic because intraspecific (ontogenetic) scaling patterns may differ from interspecific (phylogenetic) scaling patterns (and the mechanisms underlying them).

We agree and have now make the different levels of comparison and inference much clearer. First, we distinguish between inputs and outputs in ways that the earlier manuscript failed to do. Next, we clearly delineate when we are making inferences about among species patterns in within species relationships and when we are talking about within species patterns. We discuss interspecfic patterns in batch fecundity across latitudes, with a model that includes phylogenetic relatedness to correct for non-independence in observations among closely related taxa. This analysis shows that reproductive scaling increases across latitudes interspecifically. Second, for six species for which we had fecundity data spanning at least 20 degrees of latitude, we show that for all six fishes, populations at higher latitudes have a steeper reproductive scaling. (Lines 112-120, Fig. S3)

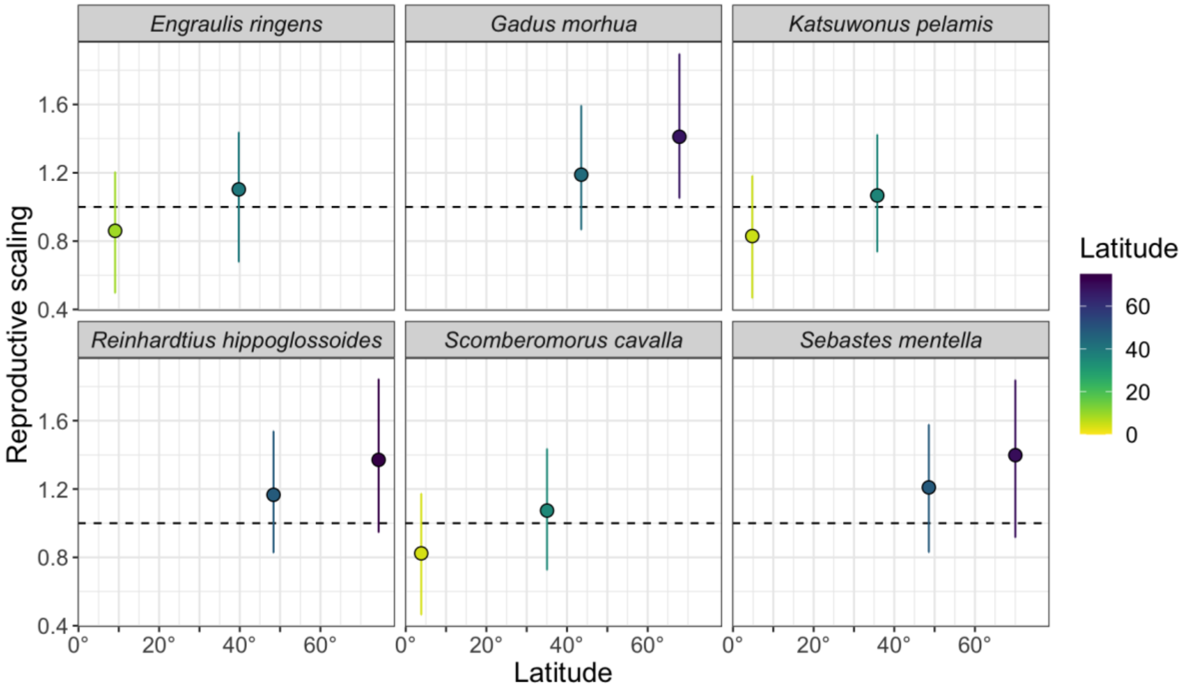

Figure S3. Intraspecific patterns in reproductive scaling. Reproductive scaling is shown for the highest and lowest latitude populations for species with reproductive data spanning >20° latitude. The error bars show the 95% credible intervals, and the colours indicate absolute latitude. The dashed black line shows reproductive isometry.

Furthermore, the life-history optimization models considered by the authors can be used to predict intraspecific scaling of reproduction with body size, but they are not obviously translatable into interspecific scaling relationships. It would be helpful if the authors separated out effects of latitude on intra- vs. interspecific scaling of the reproductive parameters that they consider.

Our models make no predictions about among species scaling relationships – for that we would need to make explicit predictions about the intercept of the intraspecific relationship between size and fecundity (and somehow account for different offspring sizes among species). We now mention this in the ms. To clarify, our model makes predictions about how intraspecific scaling should vary among species – all of our results, unless specified, relate to among species patterns in within-species scalings. (Lines 112-114)

2) The authors do not make clear that body-mass scaling parameters for reproduction may depend on the kind of reproductive output considered. Possible measures include fecundity (offspring number per clutch or litter), which they emphasize, but also total clutch (litter) mass, rate of offspring production per unit time, etc.

> *Yes, we completely agree. The reason why we used batch fecundity as the metric for fecundity is because it is the only one that is available for an appreciable number of species. (Lines 125-238, 378-380, 405-412)*

3) In my opinion, the authors oversell their claim that reproductive scaling is frequently hyperallometric. This may be true for fecundity within species of fishes (as documented by Barneche et al. 2018), but across species, clutch mass usually scales isometrically or hypometrically with maternal body mass in diverse animal and plant taxa (see e.g., Blueweiss et al. 1978; Visman et al. 1996; Hendriks & Mulder 2008; Glazier 2018). Bluewiess et al. (1978), Visman et al. (1996) and Hendriks & Mulder (2008) also report for several taxa of

animals (including fishes) and plants, that interspecific body-mass scaling exponents for fecundity are usually < 1. Similarly, Glazier (2018) reported that interspecific fecundity scaling exponents for five major taxa of crustaceans are all ≤ 1. The intraspecific scaling of clutch size in turtles is typically hypoallometric, as well (Iverson et al. 2019). Although Honěk (1993) reports that the intraspecific mass-scaling exponents for
fecundity of numerous orders of insects (the largest animal taxon) vary from 0.26 to 2.30, the common slope was 1.04 for oviparous species and 0.69 for viviparous aphids. Furthermore, a recent paper reports that fecundity declines in the largest trees (Qui et al. 2021). I could go on… I recommend that the authors do not over-generalize their results for fish fecundity to other taxa and reproductive measures.

We are specifically interested in how intraspecific fecundity scaling varies – scaling at the among species level is beyond the purview of this manuscript. We take the reviewer's point that we should not extend our generalisation beyond fish however as there are no large scale compilations of intraspecific fecundity scaling beyond fish as yet. We've modified the ms accordingly.

Specific comments:
L 17: Omit "of". The statement that "reproduction is often hyperallometric" is questionable (see also General comment #3). What measure of reproduction and what group of organisms are being considered here?

We have changed it to " Within many species, and particularly fish, fecundity does not scale with mass linearly, instead it scales disproportionately." This statement implies less generality than what we had before, while still making the point that for many taxa,

reproduction is indeed hyperallometric (2). *(Lines, 17-18)*

L 33-41: Please clarify what measure of reproductive output is being considered here. In addition, is intraspecific (ontogenetic) or interspecific (phylogenetic) body-mass scaling of reproduction being considered here? Interspecific scaling patterns of reproductive biomass are often isometric or hypoallometric, and rarely hyperallometric. For fishes, in particular, across species clutch mass scales nearly isometrically and clutch size scales hypoallometrically (see reviews by Blueweiss et al. 1978; Visman et al. 1996; Hendriks & Mulder 2008). Olsson & Gislason (2016) also report that reproductive output (g/yr) scales hypoallometrically in fishes. Note that references 1-3 cited by the authors do not provide comprehensive reviews of reproductive allometry in animals & plants, such as the ones that I mention above.

We now make clear that we are only considering reproductive output within species for fish. We also mention instances when reproduction scales hypoallometrically with mass. (Lines 116-120)

L 42-44: The papers cited (references 8-10) focus on intraspecific growth models, and do not clearly show how fecundity should scale with body mass across species. Furthermore, these models could be used to predict a variety of intraspecific scaling relationships for reproductive parameters, not just hyperallometry. The authors should make clear that these models do not explicitly predict interspecific fecundity scaling relationships, and at the intraspecific (ontogenetic) level may be used to predict a variety of scaling relationships. Overall, the specific papers that are cited do not unambiguously support the

authors' statements about reproductive hyperallometry (see also specific comments regarding each study below):

(8) Day & Taylor (1997): This commentary about intraspecific growth models makes no clear predictions about how fecundity should scale with body mass across species. Moreover, the fecundity model (equation 10) that is mentioned assumes a scaling exponent of 2/3 (see also specific comments).

Please refer to our response to point (9) below..

(9) Kozlowski (1996): This study focuses only on species showing indeterminate growth. His model groups together growth and reproduction as "production", thus not allowing a specific prediction of how reproduction should change with body size. In his model, P is assumed to scale hypometrically with body mass. The idea that allocation to reproduction may increase with body size may make sense intra-specifically, at least in some cases, but how this would translate into interspecific scaling of reproduction is not explicitly explained. Also keep in mind that the proposed model is not general, as it does not apply to determinate growers.

We disagree with the reviewer that these models do not predict reproductive hyperallometry – both models assume hypoallometric production but under all modelled conditions, the math yields hyperallometric scaling of reproduction within species (even if the text of these papers did not emphasise this result, the result was contained in the formal theory). We are specifically only interested in how fecundity scales with body ***within species****, and how that scaling differs* ***among species*** *and hence these models are most relevant.*

(10) Gadgil & Bossert (1970): In this study, changes in reproductive effort with age and body size are modelled for semelparous & iteroparous species. Although reproductive effort is predicted to increase with age in iteroparous species, their Figure 5 shows that they believe that offspring number should increase at a slowing rate or even eventually decrease after a peak is reached, as body size increases (as shown in trees: Qui et al. 2021). This represents hypoallometry, not hyperallometry. One should not confuse size with age. Furthermore, they make no specific predictions about how fecundity should scale with body size across species.

We've removed this paper as the reviewer makes a good point.

Lastly, the authors ignore the models discussed by Olsson & Gislason (2016) that predict hypoallometry of reproduction in fishes. Please explain.

Olsson and Gislasson (2016) investigates the interspecific reproductive scaling of size at maturity, which they estimate to be hypoallometric (0.84). This reproductive scaling is different to the intraspecific scaling of reproduction with female mass, which is the focus of our study. Nevertheless, we now explicitly state that interspecific relationships between mass and reproduction are likely to differ from the intraspecific (and intrapopulation) patterns examined here. (Lines 116-120)

L 57-59: Please explain why the cited models cannot predict variation in reproductive scaling.

We now say that we explicitly test the predictions of Day and Taylor and Kozlowski, that predict that life history (mortality and growth) should shape reproductive scaling. It would

be necessary to include variation in production rate parameters, season length, etc. to get predictions on variation in reproductive scaling.

L 72: Change "in" to "at".

Done.

L 84-85: Please explain. How is "reproductive scaling" quantified? This vague term is frequently used in this manuscript.

We define reproductive scaling as the mass scaling of batch fecundity. (Lines 103-106)

L 98-104: These predictions seem to make the implicit assumption that intra- and interspecific fecundity scaling relationships are the same. In other words, the intraspecific relationship of a small species should directly extrapolate into the same relationship for a larger species. Given the diversity of intraspecific relationships, this assumption seems problematic to me and should be justified.

There seems to be a miscommunication here, we're only making predictions about how within-species fecundity scalings should vary among species – we don't make any predictions about among species fecundity scaling. We've tried to make this clearer in the revised ms.

L 108-109: Are the "values of reproductive scaling" exponents (slopes)?

Yes they are log-log slopes within species. We have clarified this. (Lines 112- 120)

L 109-112. A similar increase in egg size across all individuals and species would increase the elevation of the reproductive (total offspring volume vs number) scaling relationship, but not the slope. Please clarify.

Yes, we agree and we have now corrected this.

L 250-251: Actually, Day & Taylor (1997) assume that fecundity (not just production, P) scales to the 2/3-power (equation 10), which is contrary to the claim of the authors of the submitted manuscript that the model of Day & Taylor (1997) predicts reproductive hyperallometry. The authors should make clear that it is not only the mass-scaling of P that determines the scaling of fecundity, but also how the proportion of P that is allocated to growth changes with age (which they are well aware of). In doing so, however, changes in fecundity with age may not equate with changes in size, so it seems that predicting the scaling of fecundity is not completely straightforward (see also General Comment 3).

We agree with the reviewer that, upon first glance, Eq 10 in Day and Taylor gives the impression that fecundity scales at 2/3 with mass. However, changes in energy allocation from growth to fecundity also affect the scaling, causing hyperallometry. We recognise that readers familiar with the model of Day & Taylor (1997) might also have a similar confusion, so we now have a much more detailed exposition of how the change in energy allocation alters the scaling. (Lines 320-323)

L 316: Change "use" to "used".

Done.

L 635-636: It would be helpful to specify that "reproductive scaling" (too vague) involves fecundity.

Yes, we've now clarified this.

L 882: Again, what is "reproductive scaling" in Fig. S16?

Throughout the manuscript, reproductive scaling is the slope of fecundity as a function of weight in log-log scale. We have made this clear now.

Literature cited:

Blueweiss, L., Fox, H., Kudzma, V., Nakashima, D., Peters, R., & Sams, S. (1978). Relationships between body size and some life history parameters. Oecologia, 37(2), 257-272.

Glazier, D.S. (2018) Clutch mass, offspring mass and clutch size: body-mass scaling and taxonomic and habitat variation. Chapter 3 (Pages 67-95) In: The Natural History of the Crustacea, Volume 5: Life Histories. (ed. G.A. Wellborn & M. Thiel). Oxford University Press, Oxford, UK.

Hendriks, A.J., and C. Mulder. 2008. Scaling of offspring number and mass to plant and animal size: model and meta-analysis. Oecologia 155:705-716.

Honěk, A. 1993. Intraspecific variation in body size and fecundity in insects: a general relationship. Oikos 66:483-492.

Iverson, J. B., Lindeman, P. V., & Lovich, J. E. (2019). Understanding reproductive allometry in turtles: A slippery "slope". Ecology and Evolution, 9(20), 11891-11903.

Olsson, K. H., & Gislason, H. (2016). Testing reproductive allometry in fish. ICES Journal of Marine Science, 73(6), 1466-1473.

Qiu, T., Aravena, M.C., Andrus, R., Ascoli, D., Bergeron, Y., Berretti, R., Bogdziewicz, M., Boivin, T., Bonal, R., Caignard, T. and Calama, R., 2021. Is there tree senescence? The fecundity evidence. Proceedings of the National Academy of Sciences, 118(34).

Visman, V., S. Pesant, J. Dion, B. Shipley, and R.H. Peters. 1996. Joint effects of maternal and offspring sizes on clutch mass and fecundity in plants and animals. Ecoscience 3:173-182
