# Supplementary material for: Life history optimisation drives latitudinal gradients and responses to global change in marine fishes
Source: PLoS Biol. 2023 May 25;21(5):e3002114. doi: 10.1371/journal.pbio.3002114 (PMC10212075; doi:10.1371/journal.pbio.3002114)
Supplement: Attachment — Submitted filename: 20230222_Response_to_reviewers.docx [file pbio.3002114.s026.docx]

Response to reviewers

We thank the reviewers and the editor for the constructive feedback. We address each of the comments below.

**Reviewer #1:**

The authors have addressed my questions (major and minor) pretty well. I am glad to see the newly added Fig. 3 and the text around it, which help a lot.
I only have a very minor concern here, and the authors can ignore it, if they don't agree with me. In lines 156-158, the authors said: "Our findings, in conjunction with others (11), imply that many of the life history patterns we observe in nature may be driven by the evolutionary optimisation of life histories, rather than mechanistic constraints."

I understand what they mean, and I agree with them on what they said after this sentence: "life history optimisation approaches may be a useful tool for..."

But, the first sentence sounds like evolutionary optimization and mechanistic constraints clash, but they often work in concerted ways. As the same authors said in a cited paper (Ref.11, by the way, it is a brilliant paper): "Our approach does not assume that life is unconstrained by physics and chemistry, but instead assumes that there is substantial (and underappreciated) opportunity for evolutionary optimization within these constraints."

Anyway, this is very minor, just about wording.

Congratulations on a great paper.
*Thank the kind words and for pointing out the issue with these sentences. We agree that mechanistic constraints must also affect life histories, our intention was only to highlight the role of evolutionary optimisation. We have changed the sentences to:*

*“ Our findings, in conjunction with others (11), imply that many of the life history patterns we observe in nature is driven, not only by mechanistic constraints, but by the evolutionary optimisation of life histories. At the very least, life history optimisation approaches may be a useful tool for explaining patterns in life history based only on a few key parameters.” Lines 175-178*

**Reviewer #3:**

General comments:

This revised manuscript deals with an important topic: how do reproductive scaling relationships change with latitude.  Although we already know much about how life-history traits vary with latitude in a variety of organisms, we know relatively little about how their body-mass scaling varies with latitude.  The authors have extensively revised their manuscript in many useful ways to make it stronger, but I still have two major concerns that I believe the authors should address.

(1) The authors have not acknowledged the model of Jeffery Arendt, which makes similar predictions to those presented in this manuscript (see e.g., Arendt 2011, 2015; Arendt & Fairbairn 2012).  Arendt's model is based on reported observations that size-fecundity relationships are steeper in colder than warmer environments, a pattern that is consistent with the latitudinal trend predicted and observed by the authors.  Based on these temperature-dependent size-fecundity relationships, Arendt predicts that ectotherm animals should mature later at larger sizes in colder environments (i.e., the temperature-size rule), as does the authors' model.  The predictions of Arendt's model appear to be similar to those of the authors' model, at least in part, and should be cited.  It would be useful to compare the details of these two models.  How are they similar/different?  It would also be worthwhile to point out that Arendt has found both positive (Arendt 2015) and negative (Arendt &
Fairbairn 2012) evidence supporting his model.

*Thank you for bringing these papers to our attention. The Arendt model is an interesting, alternative way of viewing the world – our model starts from the observation of higher mortality and slower growth, then predicts that maturity should be delayed and fecundity scaling should be higher under these conditions. We argue that such energy allocation patterns combined with a hypoallometric scaling of production causes an increase in fecundity scaling with higher latitudes. Conversely, Arendt’s model starts from the observation of steeper fecundity scaling in cooler temperatures and predicts that, given this pattern, it would be optimal to mature at a larger size. These are two sides of same coin if one considers temperature as the sole driver of the latitudinal patterns that we observe (which we do not). This is an important difference – Arendt’s models are entirely focused on temperature, ours is on latitudinal gradients in mortality and growth in which temperature almost certainly plays a key role. These are all important issues to discuss and we now, include a discussion of the Arendt model and compare and contrast it with ours as well as providing some suggestions on how to test these competing explanations going forward.*

(2) I am worried that the observed data show that the log-linear mass-fecundity scaling slope increases only slightly with increasing latitude (by only ~0.25), whereas the scaling slope predicted by their model changes a huge amount (by ~4.5: Figure 2) that is almost 20 times greater.  The authors point out this discrepancy between model predictions and observed data, but I feel that they should make clear that this discrepancy is huge, thus weakening one's confidence in the model.  Note also that the model predictions show that the log-linear scaling slope should be as high as 7 at high latitudes, which would indicate that fecundity increases by 7 orders of magnitude (10,000,000 times) with only a 1 order of magnitude (10 times) increase in body mass.  This seems unrealistic.  Therefore, something appears to be fundamentally wrong with (or missing from) the authors' model.  Although the authors state that it is remarkable that their model correctly predicts an increase in
the size-fecundity scaling slope with increasing latitude, this does not change the fact that it makes highly erroneous and unrealistic quantitative predictions of this change.   In a review of mechanisms affecting the temperature-size rule, Verberk et al. (2021) suggest that other environmental factors, such as resource supply, may significantly relationships between size, fecundity and temperature.  Perhaps the authors' model would be improved by focusing on the effects of specific environmental factors, such as temperature, resource supply, etc., rather than 'latitude' which represents a collage of variable environmental factors.

*We agree that the quantitative differences between our predictions and observations are, as the reviewer put it, “huge”. We now make clear that these really are major quantitative departures from the observed patterns, although some of these departures are probably the result of batch fecundity scaling being a systematic underestimate of total reproductive scaling (we now include a discussion of this issue in the ms).*

*We have different view from the reviewer that quantitative differences imply the model is wrong, but instead, as the reviewer mentions, they imply that the model is incomplete. The temptation in building any model is to engineer a perfect fit with observations by adding more parameters – the problem of course is that more parameters inevitably improve the fit, without necessarily adding real biological insights (we illustrate the particular risks of this with regards to modelling life-histories in Marshall and White 2019, TREE). Our goal here was to determine how our capacity to predict real-world life-history patterns based on the fewest parameters possible – just growth and mortality in this case. To us at least, it remarkable that we can predict any qualitative pattern based on so little and to us, it actually increases our confidence in the model.*

*Nevertheless, we firmly agree with the reviewer that the model is incomplete and currently provides a poor quantitative fit to the data. We suspect that the missing component is a realistic estimate of the overhead costs of reproduction. We now provide a simple extension of the model, which shows that predictions of reproductive scaling are much more realistic when we assume that the overhead costs of reproduction increase with body size. We also now highlight that formal empirical estimates of the overhead costs of reproduction are the next important priority to improve the predictive power of our model. We focus on this issue because we think it is the most likely universal driver of the quantitative differences between our model predictions and empirical observations. While we agree that all the other factors suggested by the reviewer might also play a role, there are no well-resolved empirical estimates of how our key predictors of growth and mortality risk covary with temperature and resource supply at a global scale. We now detail all of this in the revised manuscript.*

Specific comments:

L 201-202: In my opinion, steeper size-fecundity scaling with increasing latitude should not be considered "unexpected" because size-fecundity scaling exponents have been reported to increase with decreasing temperature in some animal species (e.g., McCabe & Partridge 1997; Scheiner & Yamplosky 1998; Weetman & Atkinson 2004; Arendt 2015; but see Arendt & Fairbairn 2012), and temperature correlates with latitude.

*We now cite these studies. Lines 202-204*

L 210-214: This is not an entirely novel prediction: see Arendt (2011). 
*We have modified the wording so that the novelty relative to Arendt (2011) is clearer. Arendt (2011) proposed that, given a high reproductive scaling, it would be favourable to delay reproduction. We make the novel prediction that delaying reproduction (e.g., because mortality is low) produces a steeper reproductive scaling due to higher relative increases in production allocated to reproduction in organisms maturing late (whenever the scaling of production is hypoallometric). The sentence now reads:*

*“Our model makes the novel prediction that fishes delaying reproduction because of low mortality produces a higher reproductive scaling.” Lines 181-183*

Literature cited:

Arendt, J. D. (2011). Size‐fecundity relationships, growth trajectories, and the temperature‐size rule for ectotherms. Evolution: International Journal of Organic Evolution, 65(1), 43-51.

Arendt, J. (2015). Why get big in the cold? Size-fecundity relationships explain the temperature‐size rule in a pulmonate snail (Physa). Journal of Evolutionary Biology, 28(1), 169-178.

Arendt, J., & Fairbairn, D. (2012). Reproductive allometry does not explain the temperature-size rule in water striders (Aquarius remigis). Evolutionary Ecology, 26(3), 745-757.

McCabe, J., and L. Partridge. 1997. An interaction between environmental temperature and genetic variation for body size for the fitness of adult female Drosophila melanogaster. Evolution 51:1164-1174.

Scheiner, S. M., and L. Y. Yampolsky. 1998. The evolution of Daphnia pulex in a temporally varying environment. Genet. Res. 72:25-37.

Verberk, W. C., Atkinson, D., Hoefnagel, K. N., Hirst, A. G., Horne, C. R., & Siepel, H. (2021). Shrinking body sizes in response to warming: explanations for the temperature-size rule with special emphasis on the role of oxygen. Biological Reviews, 96(1), 247-268.

Weetman, D., & Atkinson, D. (2004). Evaluation of alternative hypotheses to explain temperature-induced life history shifts in Daphnia. Journal of Plankton Research, 26(2), 107-116.

**COMMENTS FROM THE ACADEMIC EDITOR (lightly edited):**

I have now read in detail the authors' responses to comments from the initial version, the revised version, and both reviewers' reports. I fully share the general viewpoint that this revised version has improved a lot by nicely accounting for several problems identified in the previous version. While one reviewer is satisfied with the revised version (and only pointed out a potentially misleading sentence), the other reviewer found two major problems with this revised version.

First, previous work by Arendt, who proposed a model making a similar prediction as the authors do here (i.e. that size-fecundity relationships should be steeper in colder than warmer environments, has to be discussed as its existence might question the novelty of the authors' model.

*We fully agree and have now included a discussion on Arendt’s proposed model. Please refer to our response to reviewer 2 for more detail.*

Second, the huge overestimation of the scaling slope increase with latitude when using the author’s model (i.e. a twenty fold overestimation), which might question the consequences for quantitative predictions and practical applications in terms of management, have to be discussed as a potential limitation of this work. From my own detailed reading, I fully share these two major concerns.
*We agree that the discrepancy is very large, and we now discuss the likely sources of this discrepancy, as well as what we would need to know in order to make the model more complete. Please refer to our response to reviewer 2 for more details.*

Both issues have to be thoroughly discussed to better assess the novelty and usefulness of the model proposed by the authors. Moreover, while I appreciated the link the authors explicitly did between their findings and the existence of a slow-fast continuum of life histories, I found it too superficial and ambiguous. Thus, their findings that the pace of life of marine fish is faster at low latitudes than at high latitude matches previous findings about variation in the pace of life in relation to elevation, but is opposite to previous findings that birds have a slower pace of life in tropical areas than in temperate areas (see e.g. Wiersma et al. 2007 PNAS). A more thorough discussion of the findings reported here and the universal existence of a slow-fast continuum of life histories (at least across species within vertebrate classes) is required.

*This is a great suggestion. We now discuss how our findings differ to those in other taxa, and a possible reason for this discrepancy: in other taxa, mortality increases with latitude whereas fish experience higher mortality in the tropics than at high latitudes. It seems to us at least that mortality risk could provide a universal explanation for some of the life history patterns observed across vertebrates and that ‘latitude’ is often just a synonym for ‘mortality risk’. From this perspective, differences in in latitudinal gradients in life histories across clades are not surprising – what matters is how mortality risk covaries with latitude. We don’t raise this final point in the ms because it is perhaps too speculative, but are happy to include a brief discussion of this possibility if the editor agrees that it is worth making this point. Lines 169- 174*
