# Supplementary material for: Life history optimisation drives latitudinal gradients and responses to global change in marine fishes
Source: PLoS Biol. 2023 May 25;21(5):e3002114. doi: 10.1371/journal.pbio.3002114 (PMC10212075; doi:10.1371/journal.pbio.3002114)
Supplement: Attachment — Submitted filename: Response to editor.docx [file pbio.3002114.s027.docx]

Responses to editors

Editor

a) Please address the remaining requests from the Academic Editor (see the bottom of this letter).

*Please see our response to the academic editor for more details.*

b) We struggled a bit with the Title - normally I'd suggest something, but it's currently eluding me, so I'll pass it back to you! To me, the phrase "reproductive scaling" (dependence of fecundity on body mass?) is not very intuitive; also the study is largely about latitude and the potential consequences of climate change, neither of which are reflected here. To my mind, what the title actually means is "the dependence of fish reproduction on size ('reproductive scaling') and age ('maturation') is driven by a number of factors, but mainly latitude, with problematic consequences in the face of climate change" - could you possibly fashion a more accessible/appealing title, which encapsulates what you did, what you found, and its implications? We like titles to be declarative, with an active verb and no punctuation, if possible. I'm happy to discuss this further...!

*We have changed the title to better reflect that we are looking at life-history optimisation across latitudes and its potential implications with climate change. It now reads: “Life-history optimisation drives latitudinal gradients and responses to global change in marine fishes”.*

c) Please provide a blurb, according to the instructions in the submission form.

*Thank you- we now provide a blurb in our online submission.*

*“Life-history optimisation predicts delays in maturity with decreasing mortality, altering the size-fecundity relationship. For marine fishes, these predictions match observed latitudinal gradients, with a delayed maturity and steeper increases in fecundity with body mass at high latitudes.”*

d) Please address my Data Policy requests below; specifically, we need you to supply the numerical values underlying Figs 1AB, 2ABC, 3C, 4AB, S1ABC, S2ABC, S3, S4ABCDEF, S5ABCD, S6ABCD, S8, S9ABCD, S10ABCD, S11ABC, S12, S13AB, S14, S15, either as a supplementary data file or as a permanent DOI'd deposition. I note that you already have a zipped supplementary data folder, but the data in these files looks rather "raw" to me, and we do need the values displayed in the Figs and/or any code needed to generate them.

*We have specified numerical values were applicable and made it clear when figures were generated by life-history model simulations rather than statistical fits.*

e) Please cite the location of the data clearly in all relevant main and supplementary Figure legends, e.g. "The data underlying this Figure can be found in S1 Data" or "The data underlying this Figure can be found in <https://aus01.safelinks.protection.outlook.com/?url=https%3A%2F%2Fdoi.org%2F10.5281%2FXXXXX&data=05%7C01%7Cm.alvareznoriega%40aims.gov.au%7C418fcba33fd043a0ddd108db2b0ad82e%7Ce054a73b40dc4ae39fce60c537aa6fac%7C0%7C0%7C638151097812423430%7CUnknown%7CTWFpbGZsb3d8eyJWIjoiMC4wLjAwMDAiLCJQIjoiV2luMzIiLCJBTiI6Ik1haWwiLCJXVCI6Mn0%3D%7C3000%7C%7C%7C&sdata=i%2BDITTjlcwViUvii77ZaFmEqnwM6XSPWfOANVq4%2B98o%3D&reserved=0>"

*We now specify which data file corresponds to each figure.*

Academic editor

This revised version nicely accounts for all remaining problems one reviewer and myself identified in the previous version. In particular, the revised MS provides both a thorough discussion of the model proposed by Arendt and a convincing explanation (involving the metric used to measure the reproductive output and confounding effect of reproductive costs) for the mismatch between the expected and observed scaling coefficients.

*We are pleased that the revised manuscript was well received.*

I also enjoyed the new and more thorough discussion about the slow-fast continuum of life histories. However, although it is still speculative, I found the authors' interpretation that mortality risks rather than latitude per se drive life history covariations highly interesting and worth explicitly presenting in this work (after lines 160-163).

*We are pleased that the academic editor found our hypothesis interesting.*

In addition I found the following details to fix:

l. 132: "allocation", not "investment"

*Done.*

l. 136: "allocated", not "invested"

*Done.*

l. 161: "are driven" instead of "is driven"

*Done.*

l. 182: "allocation", not "investment"

*Done.*
